# BiP Clustering Facilitates Protein Folding in the Endoplasmic Reticulum

**DOI:** 10.1371/journal.pcbi.1003675

**Published:** 2014-07-03

**Authors:** Marc Griesemer, Carissa Young, Anne S. Robinson, Linda Petzold

**Affiliations:** 1 Department of Applied Mathematics, University of California, Merced, Merced, California, United States of America; 2 Department of Chemical Engineering, University of Delaware, Newark, Delaware, United States of America; 3 Department of Chemical and Biomolecular Engineering, Tulane University, New Orleans, Louisiana, United States of America; 4 Department of Computer Science, University of California, Santa Barbara, Santa Barbara, California, United States of America; University of Houston, United States of America

## Abstract

The chaperone BiP participates in several regulatory processes within the endoplasmic reticulum (ER): translocation, protein folding, and ER-associated degradation. To facilitate protein folding, a cooperative mechanism known as entropic pulling has been proposed to demonstrate the molecular-level understanding of how multiple BiP molecules bind to nascent and unfolded proteins. Recently, experimental evidence revealed the spatial heterogeneity of BiP within the nuclear and peripheral ER of *S. cerevisiae* (commonly referred to as ‘clusters’). Here, we developed a model to evaluate the potential advantages of accounting for multiple BiP molecules binding to peptides, while proposing that BiP's spatial heterogeneity may enhance protein folding and maturation. Scenarios were simulated to gauge the effectiveness of binding multiple chaperone molecules to peptides. Using two metrics: folding efficiency and chaperone cost, we determined that the single binding site model achieves a higher efficiency than models characterized by multiple binding sites, in the absence of cooperativity. Due to entropic pulling, however, multiple chaperones perform in concert to facilitate the resolubilization and ultimate yield of folded proteins. As a result of cooperativity, multiple binding site models used fewer BiP molecules and maintained a higher folding efficiency than the single binding site model. These *insilico* investigations reveal that clusters of BiP molecules bound to unfolded proteins may enhance folding efficiency through cooperative action via entropic pulling.

## Author Summary

The misfolding of proteins carries important implications for diseases such as Alzheimer's, Parkinson's, cancer, and diabetes. Once misfolded, proteins tend to associate into aggregates that pose a toxic threat to the cell. Chaperones are proteins that rescue the cell from an accumulation of these maladjusted proteins through dissociation of toxic oligomers and proper (re)folding. The endoplasmic reticulum (ER) is an organelle that serves as the staging ground for the chaperone activities of protein transport, folding, and maturation in the early secretory pathway. We have developed a computational model to investigate potential mechanisms that enable multiple ER-resident molecules working in concert to effectively fold peptides and transport nascent proteins across the ER membrane. Although previous models focused on chaperone interactions with peptides, we have explored the influence of cooperativity among chaperone molecules to assist in protein folding and maturation. We found that chaperone cooperation led to a higher yield of folded molecules compared to when chaperones bound to peptides in a 1∶1 stoichiometry. We have concluded that the clustering or multiple binding of chaperones may facilitate protein folding *in vivo*.

## Introduction

Protein homeostasis, or proteostasis, is characterized by the integration of biological pathways that modulate protein biogenesis, maturation, transport, and degradation. As a critical element to cell survival, networks of molecular chaperones, foldases, and quality control components minimize the effects of cell stress in order to revert to a homeostatic environment [1]. Proteostasis occurs in distinct subcellular environments and is constantly monitored by stress-signaling pathways. In eukaryotes, the endoplasmic reticulum (ER) is the first membrane-enclosed organelle of the secretory pathway, which ascertains the fidelity of protein folding, maturation, biogenesis (*i.e.* translation and ER translocation), and ER-associated degradation (ERAD). In the yeast *S. cerevisiae*, multiple ER quality control mechanisms have been identified to modulate these critical ER processes, including associated chaperone/co-chaperone interactions. Specifically, molecular chaperones dissociate aggregates, self-associating conglomerations of unfolded and misfolded proteins, which would otherwise interfere with the cell homeostasis leading to cell dysfunction and death [2]. Despite the ubiquitous nature of molecular chaperones, a variety of insults can overwhelm the ER's processing capacity including nutrient deprivation, pathogenic infection, cell differentiation, or alterations in calcium concentration or redox status. As a consequence of ER stress, aberrant proteins accumulate within this organelle, triggering intracellular pathways collectively referred to as the unfolded protein response (UPR). In eukaryotes, the UPR transcriptionally up-regulates genes encoding molecular chaperones [3], ERAD machinery [4]–[7], key enzymes of lipid biosynthesis [8], and other components of the secretory pathway [9]–[11]. Notably, several key features of the UPR are conserved across eukaryotes; although expanded in scope, the mammalian UPR has similar attributes to that of *S. cerevisiae*, particularly with respect to the Ire1p-dependent regulation of unfolded proteins and BiP modulation of the response (reviewed [12]). The elucidation of these pathways – specifically the interplay between UPR and ERAD – has become of growing importance in therapeutics as loss of proteostasis has been suggested to lead to a number of human diseases including Alzheimer's, Parkinson's Disease and Type II Diabetes [13].

In the early secretory pathway, protein fidelity is attributed to select chaperone/co-chaperone interactions (Hsp70 and Hsp40 proteins, respectively) conserved via evolution from yeast to humans. As one of two distinct Hsp70 molecular chaperones in the ER, BiP/Kar2p binds preferentially to hydrophobic residues of nascent or unfolded proteins [14], [15]. BiP, the yeast homolog of binding protein immunoglobulin (referred to as Kar2/Grp78 [16]), has been identified as an essential component of ER translocation, protein folding and maturation, karyogamy, and ERAD [17]–[20]. To facilitate protein folding, co-chaperones stimulate the binding of BiP to substrates whereas nucleotide exchange factors (NEFs) assist in BiP's stochastic release via cycles dependent upon adenosine triphosphate (ATP). For example, co-chaperone Sec63 directly interacts with BiP, increasing its affinity for nascent proteins as they advance through the translocation pore in *S. cerevisiae* [21]–[23]. In yeast, the posttranslational translocation of nascent peptides is mediated by a heptameric Sec complex, composed of Sec63, Sec62, as well as the heterotrimer Sec61, which serves as the protein-conducting channel [24], [25]. Photo-cross-linking experiments have shown that nascent peptides are in continuous contact with Sec61 during protein translocation [26]. More recently, cryo-electron microscopy established that a single Sec61 heterotrimer enables the progress of nascent proteins across the ER membrane, a conserved feature manifested in both yeast and mammals [27]. In addition to ER translocation, BiP's interaction with co-chaperone Scj1 has been implicated in protein folding and maturation [28], [29], and degradation of aberrant proteins [30].

ER translocation, protein folding and maturation, as well as ERAD are conserved mechanisms across eukaryotes. As such, the model eukaryotic organism, *S. cerevisiae*, provides an effective experimental platform to elucidate an improved mechanistic-understanding of proteostasis, specifically with regards to ER chaperone/co-chaperone interactions. Proteomic studies have identified absolute levels of protein expression and verified the location of ER-resident proteins [31], [32]. These data suggest that the ER-resident chaperone BiP exceeds the level of all co-chaperones in the ER by at least an order of magnitude at conditions of normal growth, and is significantly up-regulated during the UPR [6], [33] indicating a significant increase in BiP's total abundance. Furthermore, BiP binds to substrates with varying affinities [14], suggesting BiP responds to the protein's folding requirements. Interestingly, from an experimental perspective, the spatial localization of ER-resident chaperones or co-chaperones has been evaluated for only Sec63 during the process of translocation. In yeast, membrane protein Sec63 must by necessity be localized at the ER membrane in order for nascent proteins to translocate [34]. Collectively, this evidence suggests that BiP's spatiotemporal profile may be a contributing factor to its diversity and functionality in the ER. This hypothesis was previously posited and computationally explored [35]. Those results were in agreement with Sec63 experimental results, and further suggested that BiP clusters may exist in order to facilitate the efficient translocation of nascent proteins.

That BiP performs disparate functions owes to its tendency to bind many different types of proteins. Binding of multiple chaperones to unfolded proteins has been established and determined to be kinetically favorable [36]. The transport of nascent proteins into the ER involves many BiP molecules working in concert. Algorithms to predict binding sites have been developed, and there are many examples of proteins that have repeated hydrophobic stretches of amino acids [15], [37], [38], which predict the presence of multiple binding sites. Aggregates have also been found to have the analogous binding sites [39], while their large size implies that multiple BiP molecules could engage them at individual sites simultaneously. Here, we refer to clustering as the process by which multiple BiP molecules bind to individual binding sites that can be predicted from hydrophobic residues along the length of the protein. This is in contrast to aggregation, where self-associating conglomerations of unfolded and misfolded proteins combine into larger toxic structures.

Experimental evidence has revealed that the refolding of misfolded proteins and aggregates occurs in the presence of a molar excess of chaperones [40], which led investigators to propose that multiple chaperones apply a cooperative stretching force known as entropic pulling [41], [42]. The random motion of several bound individual chaperones on a peptide can sum up to an effective unfolding enabling re-initialization of the folding process. The additional molecules provide an inertial brace that stabilizes the interaction between chaperone and protein. In the case of chaperone-mediated disaggregation, the brace is the aggregate itself. A similar mechanism enables chaperones to assist nascent peptides during ER translocation.

Cooperative action underlies many cellular processes including signal transduction [43], protein transport [44], and chemotaxis [45]. In the chaperone system, binding is not cooperatively enhanced. Rather, the rate of solubilization and renaturing of proteins increases with the number of chaperone molecules [40].

In this study, we created a computational model to investigate the extent that an ER-resident chaperone, BiP — spatially localized to “clusters” — may influence the extent of protein folding. Our model includes BiP, the co-chaperones Scj1 and Sec63, and multiple states corresponding to unfolded proteins and complexes. This work implements ER-resident chaperone/co-chaperone interactions, experimental insights [21], [46]–[48], estimates of species concentrations determined for *S. cerevisiae* [32], and binding affinities between BiP, Sec63, and synthetic peptides [46], . When experimental data were not available, established estimates from the mammalian literature for these highly conserved mechanisms and proteins were used (Text S1, Table S1, Supporting Information). To assess the potential advantages of clusters, this model was used to evaluate the extent to which gradients of BiP molecules may facilitate its activities in protein folding and aggregate disassembly. Previous models [49]–[59] have included varying aspects of chaperone activities and interactions, yet only accounted for a single binding site scenario; in contrast, our model focuses on multiple binding as the mechanism to facilitate BiP's roles in the ER.

This study provides a detailed analysis of (i) the quantitative impact of chaperone clustering activity in the ER and contributing factors leading to efficient protein folding; and (ii) the potential mechanisms and interplay among components of ER quality control. Together, this framework provides an improved mechanistic understanding of chaperone/co-chaperone interactions, as well as possible strategies to minimize the accumulation of misfolded proteins.

## Models

### Model Description

**Figure 1:**
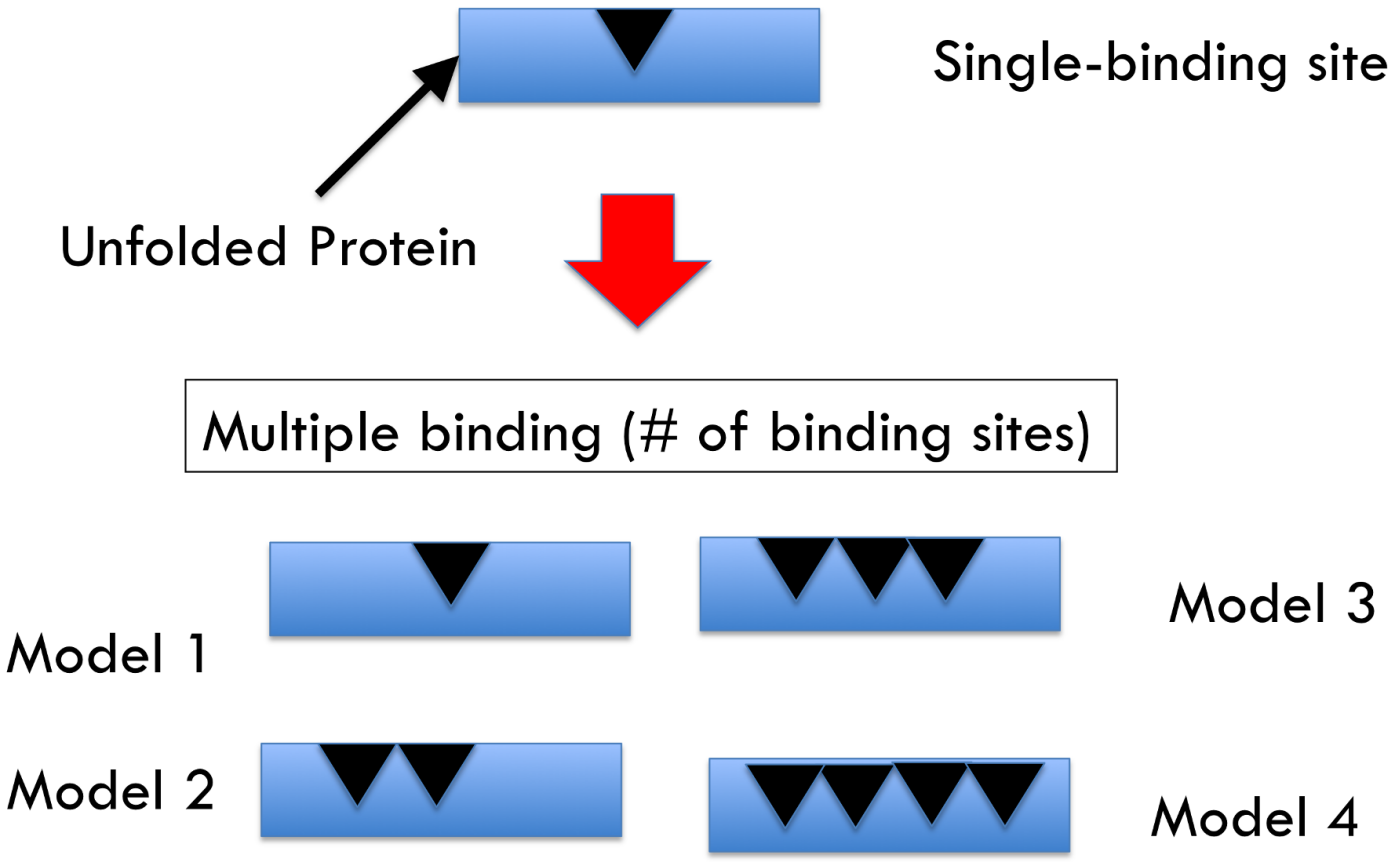
Models defined by number of binding sites. Schematic of the 4 models defined by the number of binding sites.

**Figure 2:**
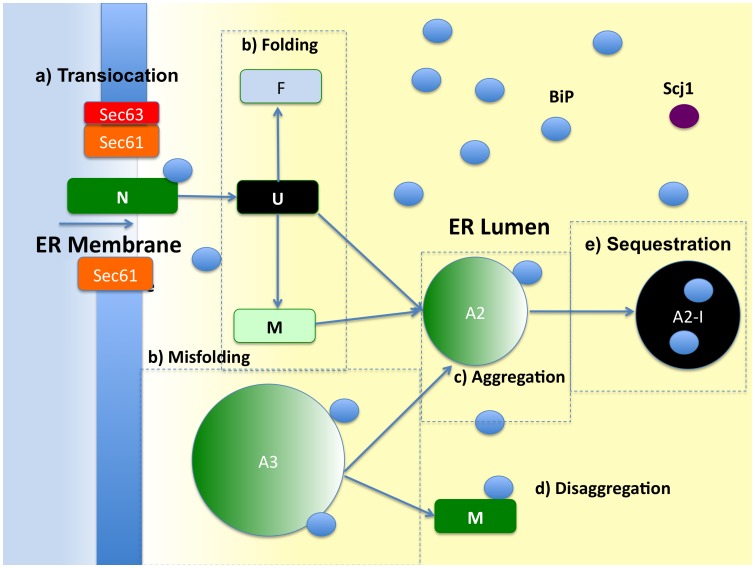
Schematic of the processes in the ER and their spatial location. Protein translocation occurs at the ER membrane, while the other processes can occur in the ER lumen. Processes include: (a) translocation; (b) folding/unfolding/misfolding; (c) aggregation; (d) disaggregation; (e) sequestration. Species are represented as follows: nascent protein (N); folded protein (F); unfolded protein (U); misfolded protein (M); BiP; Translocation Pore (Sec61); Sec63; size 2 aggregate (A2); size 3 aggregate (A3); size 4 aggregate (A4).

**Figure 3:**
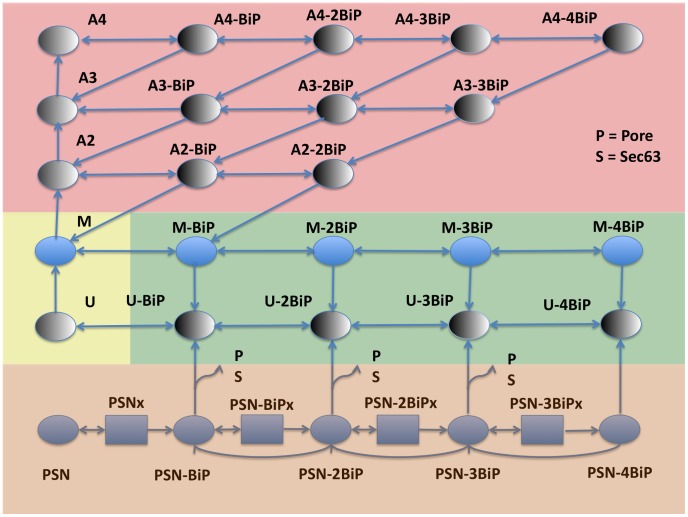
Schematic of the ODE model. Background colors represent translocation (orange), unfolding (green), misfolding (yellow), and aggregation/disaggregation (red) modules. Additional states and reactions involving the luminal co-chaperone Scj1 are accounted for in the aggregation, unfolded, and misfolded modules, but are omitted from this diagram due to space limitations. Species are represented as follows: Pore; nascent protein (N), sliding state (x); folded protein (F); unfolded protein (U); misfolded protein (M); BiP; Sec63; size 2 aggregate (A2); size 3 aggregate (A3); size 4 aggregate (A4). Sliding states (x) mimic the movement of the nascent protein further into the lumen.

We created an ordinary differential equation (ODE) model to study the efficiency of protein folding due to the molecular heterogeneity of ER-resident chaperone, BiP. Four sub-models were created that differ by the stoichiometry of binding sites to the protein species: one, two, three, and four (as shown in Figure 1). To evaluate model performance two metrics were accounted for: (i) folding efficiency (*i.e.*, fraction of proteins folded); and (ii) chaperone cost (*e.g.*, the molecular resources needed to achieve a specified level of efficiency). A schematic of the ER, as well as prominent protein-protein interactions, is shown in Figure 2. A total of 60 species and 125 reactions are evident in the largest model. Within a model, numerous states have been depicted in Figure 3. A comprehensive list of all reactions, states, rates and initial conditions is referenced in the Supporting Information. The initial units of species abundance were converted to concentration by incorporating an ER volume of 0.7 µm^3^ [60]. Model parameters were obtained from literature sources (where available), as detailed in the Supporting Information (Text S1, Tables S1–S7).

### Model Structure

Our model monitored the fate of soluble proteins within the ER lumen by investigating the composition of six modules, as follows:

1. Protein Synthesis and Translocation
  A nascent protein (N) is synthesized by a ribosome localized on the ER membrane (cytosolic interface) near the translocon, as shown in Figure 2a. Sec61 channels, referred to as translocation pores, are activated by the binding of co-chaperone Sec63, and nascent proteins can then start the process of translocation through the ER membrane. In this study, we modeled the movement of nascent proteins across the ER membrane as post-translational translocation, which directly involves the Sec complex of eukaryotes. The nascent protein progresses forward into the pore channel (Pore-Sec63-N→Pore-Sec63-N*x*), exposing a binding site (*x*) within the lumen where a BiP molecule may bind. We assume that a BiP molecule preferentially binds at a site closest to the membrane, consisting of hydrophobic residues, as the co-chaperone/chaperone interaction facilitates this binding (Pore-Sec63-N*x*+BiP→Pore-Sec63-N-BiP). Subsequently, a nascent protein can irreversibly proceed into the lumen, thus exposing a second binding site to BiP at the channel; however, it cannot assume the first configuration that consisted of the initial BiP at the membrane, since BiP acts as a “stopper” to prevent this motion. This cycle continues until the nascent protein has completely exited the channel. The nascent protein then dissociates from the Pore-Sec63 complex and is now classified as an unfolded protein (U), with bound BiP molecules spaced intermittently along the length of the peptide. In this model, the Pore-Sec63 complex disassociates into its constituent proteins, yet whether Sec61 pores are involved in successive rounds of ER translocation is unclear [61]–[63].
2. Misfolding, Unfolding, and Productive Folding
  This multifaceted pathway is detailed in Figure 2b. In this scenario, the default initial protein state is unfolded, but may terminally misfold, a circumstance dependent on the ratio of unfolded proteins to chaperones. Misfolded proteins cannot spontaneously progress towards an unfolded state since a key role of chaperone/co-chaperone systems is to bind to misfolded proteins and unfold them, thereby resulting in an opportunity to fold to its proper confirmation. In this model, the mechanism of the chaperone system begins with unfolded or misfolded proteins binding to the J-type co-chaperone Scj1 (analogous to Erj3 in humans) or with BiP forming binary complexes (Scj1∶U/M or BiP∶U/M, where the “/” indicates “either-or”). BiP binds weakly to substrates while Scj1 accelerates ATP hydrolysis to facilitate BiP's conformational change, thus an increased affinity between BiP and the unfolded protein. Consequently, BiP and Scj1 may act synergistically. BiP molecules bound to U/M passively prevent misfolding and aggregation (or in the case of aggregates, further oligomerization). Unfolded proteins fold either spontaneously or by chaperone assistance [64]–[67].
3. Aggregation
  Aggregation is illustrated in Figure 2c. Our model describes aggregation as a process in which non-native proteins associate and evolve by the addition of unfolded or misfolded proteins. Aggregation by this process could lead to large masses of hundreds of monomers, which as a model would be intractable computationally. Thus, we limited the size of aggregates to four. Notably, larger aggregates require the assistance of additional ERQC components other than BiP and Scj1 alone [68], limiting applicability here. Each aggregate maintains a number of binding sites up to the size of the aggregate and/or the number of binding sites of the model, whichever is less. Thus, the single binding site model has only one binding site even for a size four aggregate (A4), while the four site model has four binding sites on A4. The rate of accumulation of proteins into aggregates is assumed to be equal for all sizes of aggregates (10^7^ M^−1^ s^−1^), except the first step (nucleation) which was constrained as one-tenth of all sequential steps, thus rate-limiting [69]. We assume that the aggregation is irreversible except through the action of chaperones. To substantiate this assumption, Diamant *et al.* [68] demonstrated that Hsp70 chaperones have a diminished ability to re-solubilize very large aggregates by themselves.
4. Disaggregation
  Disaggregation is shown in Figure 2d. Disaggregation is critical for the recovery of ER homeostasis following the accumulation of protein aggregates due to classical cell stress responses, such the heat shock response or UPR. Successful disaggregation leads to a misfolded monomer bound to a single chaperone, while the remaining chaperones and aggregate exist in a complex [A_j-1_-iBiP where j-1 is the new aggregate size and i is the number of BiPs still bound to the aggregate. We have assumed that aggregates do not dissociate freely, in the absence of chaperone interactions [70], [71]. However, chaperones can extract a constituent misfolded protein from the aggregate, reducing the aggregate size. In an iterative manner, this process yields total disaggregation. Scj1 facilitates BiP's function by binding initially to the aggregate before ATP hydrolysis, thus securing the ER-resident chaperone, BiP, to the substrate [72]. Chaperone/co-chaperone interactions stabilize the aggregate at its current size. As with the folding or triage reactions, BiP can perform disaggregation independent of Scj1, but at a lower binding rate. We set the disaggregation rate to 1 s^−1^ for BiP-only mediated reactions.
5. Sequestration
  Aggregates can become insoluble, inert bodies (I). We assumed a single rate of irreversible entrapment for all aggregated species. Chaperones are lost, as they become entangled in these structures and eventually degrade by ERAD. We set this insoluble rate to 1 s^−1^ [59]. Figure 2e illustrates this process.

#### Cooperativity

Cooperativity is modeled as entropic pulling, via mass-action reactions, and highlighted in the example reactions below (see Tables S2–S7 in Text S1 for the full description),
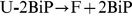
(1)

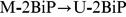
(2)

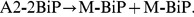
(3)


### Model Metric Equations

In this study we assessed two model metrics: folding efficiency and chaperone cost. The former is given by the total number of folded proteins at the end of the simulation divided by the total number of unfolded proteins (yielding a fractional range between zero and one),
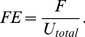
(4)Chaperone cost is defined as the average number of bound chaperones per unfolded protein per unit time. This metric combines the time spent on the protein with the total number of chaperones bound at the end of the simulation,
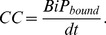
(5)


## Results

### Model Results

**Figure 4:**
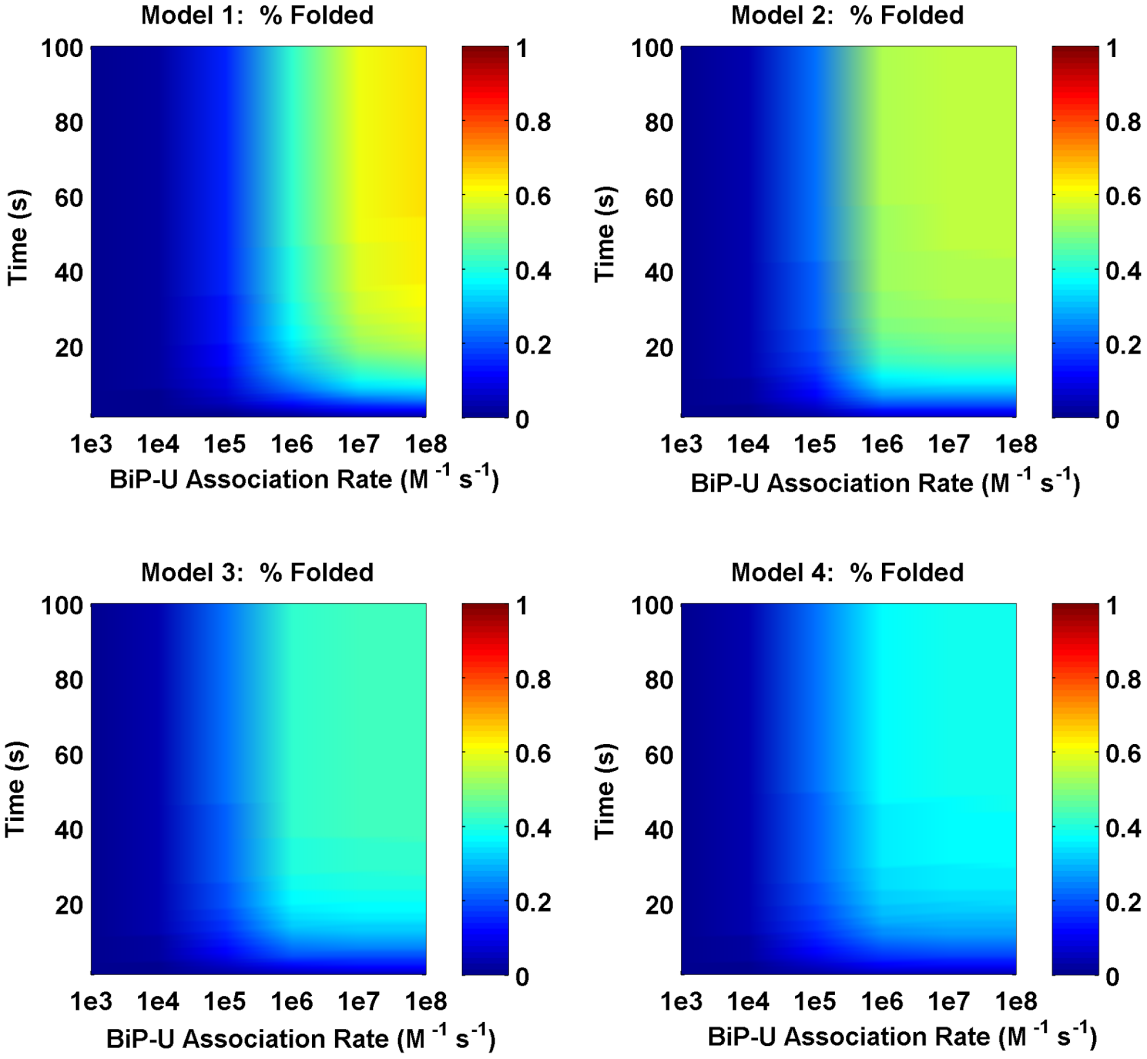
Folding efficiency vs. BiP binding rate without cooperativity. Comparison of the folding efficiency (*i.e.* fraction of proteins folded) as a function of the binding rate between BiP and unfolded proteins. In this scenario, there is no cooperative effect among chaperones in folding, unfolding, or disaggregating proteins. The model number refers to the number of binding sites. In this scenario, Model 1 has the highest folding efficiency, followed by Models 2, 3 and 4.

**Figure 5:**
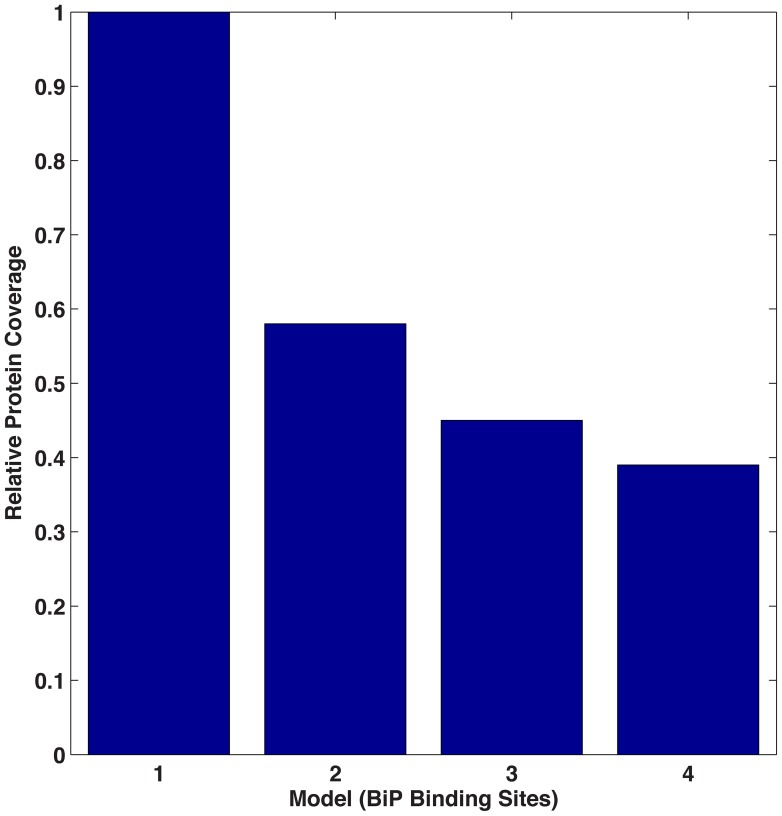
Relative protein coverage. Protein coverage of the four models relative to Model 1 in the noncooperative scenario. Coverage refers to the percentage of proteins that are protected from misfolded and aggregation at any one time.

**Figure 6:**
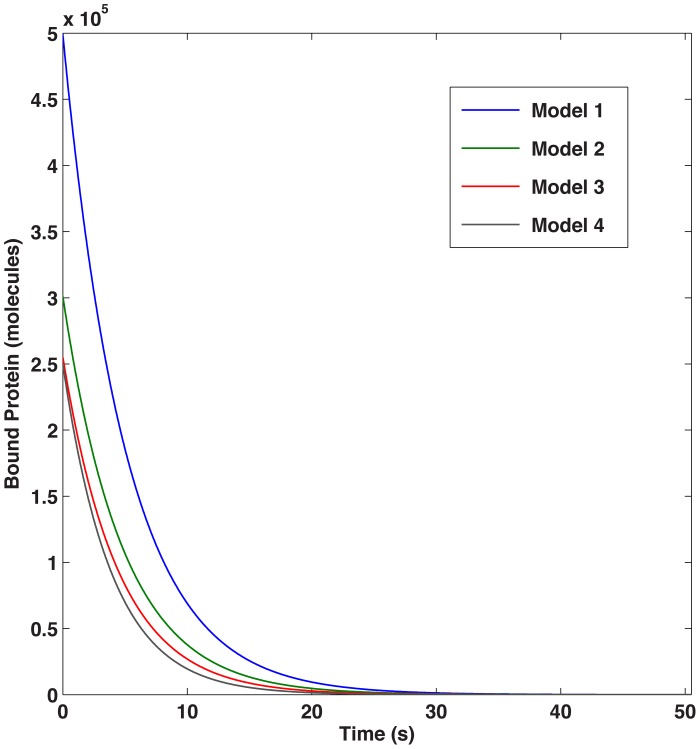
Protein coverage. Time series of the amount of bound protein for the different models, showing greater coverage for the single binding site model.

Steady-state solutions for the four model cases (corresponding to 1, 2, 3 or 4 binding sites) were completed for different values of BiP association to unfolded proteins. Figure 4 compares the models in terms of folding efficiency (*i.e.*, total folded as a percentage of total protein) and association rate. In the absence of cooperativity, the single binding site model (Model 1) yields increased levels of folded protein, as unfolded protein binding sites are more easily saturated, providing more BiP coverage of the unfolded protein population (Figure 5). When one examines the time of interaction between BiP and unfolded proteins, BiP covers more proteins, each for a longer period of time (in protein per second) as compared to the alternative models (Figure 6). This effect occurs at low ratios of BiP∶U, hence the chaperone is classified as a ‘holdase’ [73]. However, the simpler non-cooperative models are incomplete in describing the entropic pulling data [40], hinting that multiple BiPs must also act as a cooperative ‘unfoldase’, in line with previous observations [73].

In comparison to other models herein, the degree of folding in the single binding site model is more highly dependent on the association rate (Figure 4). Multiple BiP binding events minimize the potential of an unfolded protein towards either misfolding or aggregation pathways, as a consequence of redundant binding events. We have not accounted for ATP molecules in our simulations, since this aspect would only be of concern in a depleted ATP environment [70].

**Figure 7:**
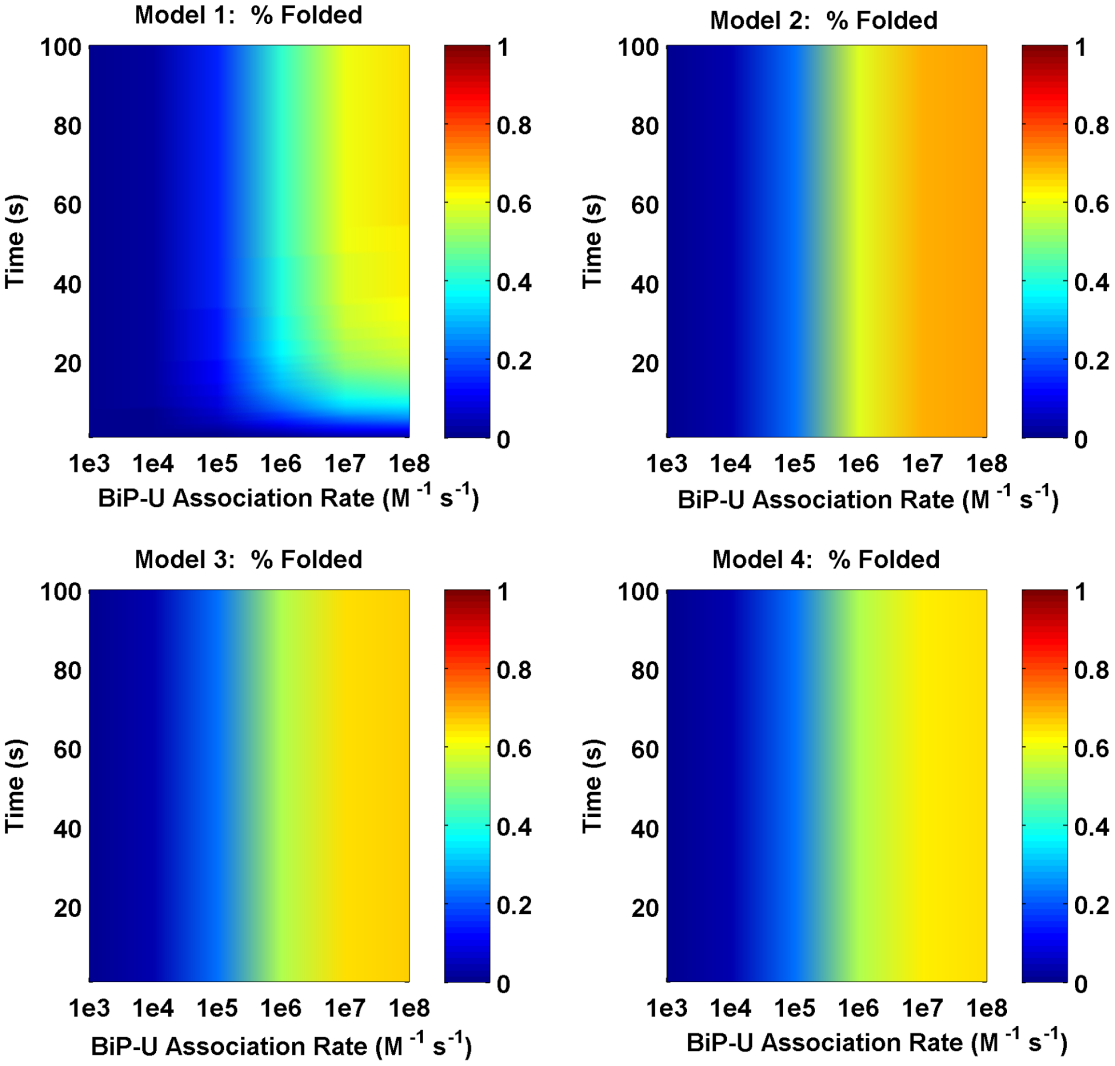
Folding efficiency vs. BiP binding rate with cooperativity. Comparison of the folding efficiency (*i.e.* fraction of proteins folded) as a function of the binding rate between BiP and unfolded proteins with a cooperativity factor of C = 10. In this scenario, Model 2 has the highest folding efficiency.

**Figure 8:**
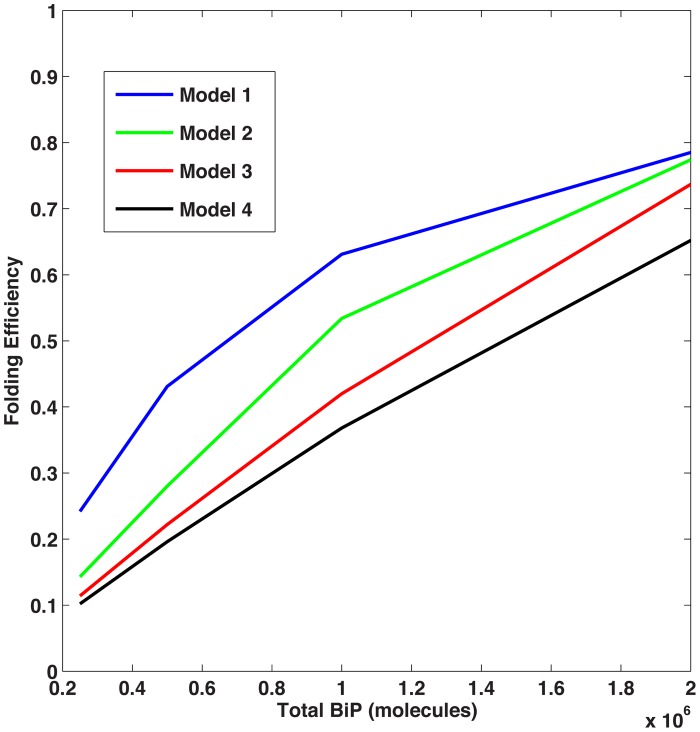
Folding efficiency vs. cooperativity. Folding efficiency of the four models as a function of the cooperativity factor.

In line with the entropic pulling contributing to BiP function, we increased the rates of folding, unfolding, and disaggregation by a factor of C, to reflect the cooperativity of multiple chaperones participating in these select intracellular activities. With C = 10 (*e.g.*, the lower end of the range (1–100) reported in the literature [41], Model 2 resulted in the highest level of folded protein. Less folding was observed in Models 3 and 4 as compared to Model 2 since coverage competes with cooperativity (Figure 7). When cooperativity is implemented, the folding efficiency for Models 2, 3, and 4 increases; Model 2 performs optimally for C>5, as shown in Figure 8.

**Figure 9:**
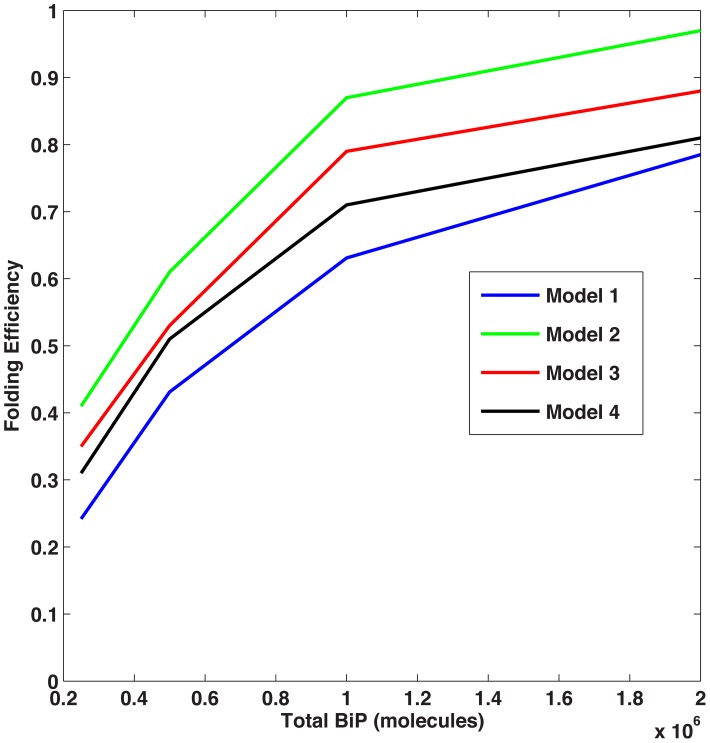
Folding efficiency vs. number of BiP molecules. Comparison of the folding efficiency as a function of the number of BiP molecules with no cooperativity and U = 1.0 · 10^6^ molecules. In this scenario, Model 1 folds most efficiently.

**Figure 10:**
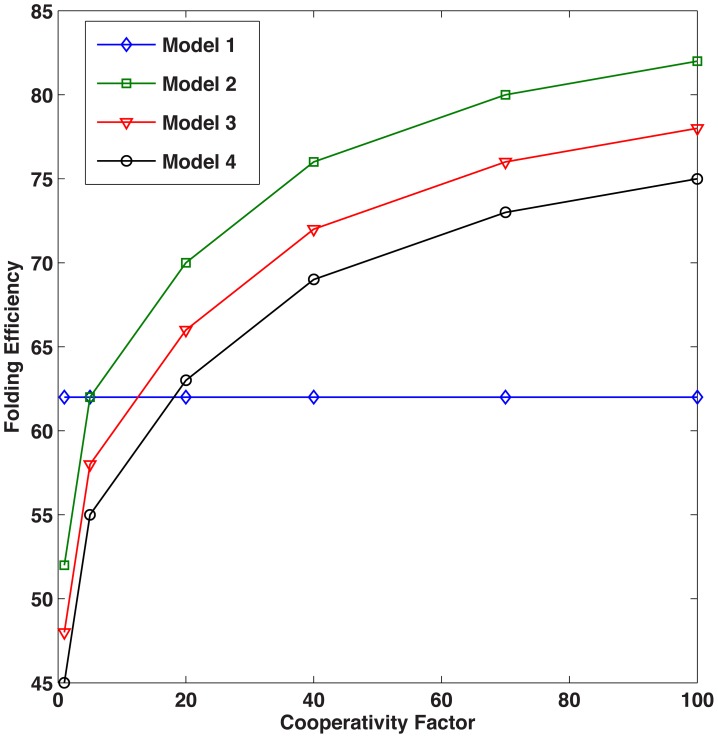
Folding efficiency vs. number of BiP molecules, cooperative scenario. Folding efficiency of the four models as a function of BiP concentration with cooperativity factor C = 10. In this scenario, Model 2 folds most efficiently.

We then varied the concentrations of total BiP and unfolded protein to examine the effect on the two metrics described previously. As expected, increased concentrations of BiP led to higher levels of folding and less aggregation. Unexpectedly, we discovered that the ratio of BiP∶U is a more important factor than the concentrations of either species alone. In the noncooperative scenario, Model 1 produced the most folding (Figure 9); however, when cooperativity was added, Models 2–4 attained higher folding efficiencies (Figure 10). These results suggest that when the BiP∶U ratio is low (*e.g.*, conditions of ER stress), cooperativity provides an advantage for multiple binding. At higher BiP concentrations (*i.e.*, relative to the concentration of U), cooperativity became a factor of less importance since the majority of unfolded proteins were protected from aggregation. As a result, more binding sites were occupied, leading to an equalization in the total amount of folding among the four models, *i.e.* the cooperativity effect was less pronounced.

**Figure 11:**
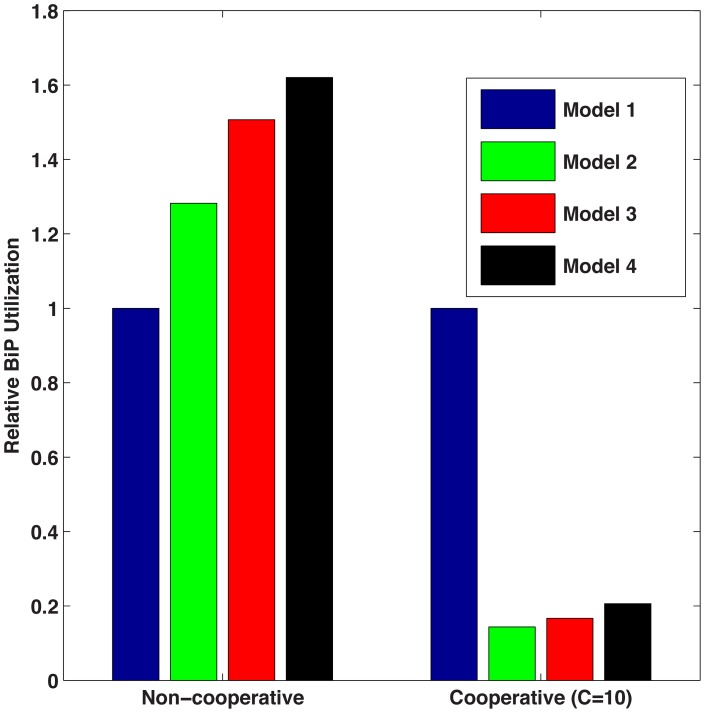
Chaperone cost. Comparison of the BiP cost of the four models for both non-cooperative and cooperative scenarios. It is better to have lower chaperone cost so that fewer chaperones are required.

Figure 11 shows that chaperone cost (*i.e.*, average chaperones bound per second compared to unfolded, misfolded and aggregated proteins) decreased substantially for Models 2, 3 and 4 in comparison with Model 1, as shown for the cooperative case. In general, it is better to maintain a lower cost metric resulting in fewer chaperones bound per second. Due to the faster rates of disaggregation, unfolding, and folding in the cooperative scenario for Models 2, 3 and 4, chaperones maintained a shorter interaction with proteins. More chaperones were engaged with a single protein in Models 2–4, yet this result was counteracted by decreased time that chaperones were bound to the protein.

### Parameter Correlation Study

**Figure 12:**
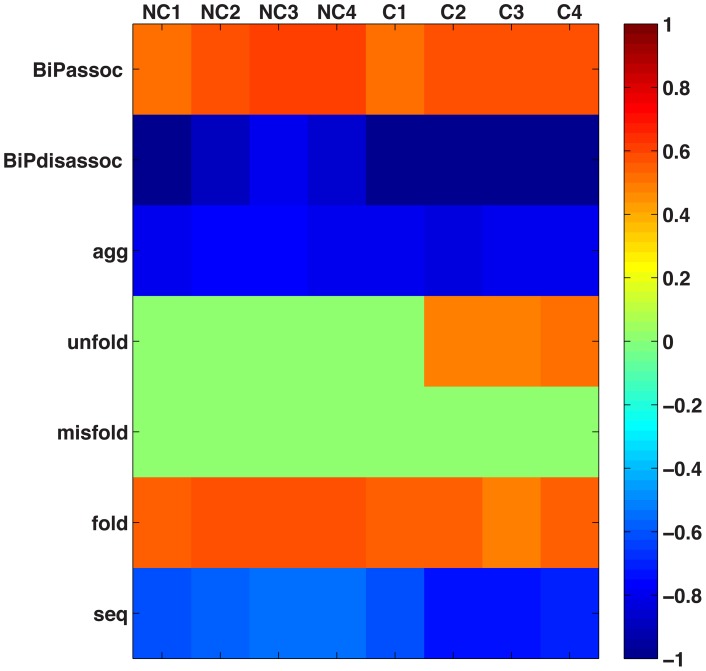
Parameter map. Map of the parameter study indentifies effects of varying 7 parameters with respect to protein folding efficiency.

To investigate the correlation between parameters and folding efficiency for the different models and cooperativity scenarios, a heatmap is shown in Figure 12. In this study, we varied BiP's association rate, the aggregation rate from 10^3^ to 10^8^ M^−1^ s^−1^ and varied the folding, unfolding, misfolding, BiP disassociation, and sequestering rates from 10^2^ to 10^−3^ s. Over these six orders of magnitude, the folding efficiencies were recorded then correlations were completed between parameter ranges and folding efficiencies. Note: this analysis varied one parameter at a time, while keeping the others constant.

1. The BiP-U association rate has a positive correlation with the folding efficiency of 0.5–0.6 for all models and cooperativities. Thus, although the folding efficiencies are different for the four models and cooperativity scenarios, the increases in efficiency are proportional to each other. The recruitment of BiP to proteins is also enhanced by the co-chaperone Scj1 interactions. The medium correlation most likely occurs as a consequence of minimal BiP molecules bound to a fraction of unfolded proteins, *i.e.* the coverage effect.
2. The BiP-U disassociation rate is highly anti-correlated with the folding efficiency (∼−0.99). When the disassociation rate is low, BiP remains bound to the protein (or aggregate), thus allowing for additional time for triage and ultimately folding.
3. The aggregation rate is negatively correlated with the folding efficiency for all models and scenarios. This effect is directly dependent upon folding and aggregation, processes that are in kinetic competition.
4. For the non-cooperative scenario, the unfolding rate is uncorrelated with the folding efficiency across six orders of magnitude. In contrast, chaperones increasingly impact unfolding and maintain a positive correlation with respect to folding yield, within the cooperative scenarios. Since chaperones are involved, the BIP-U association and disassociation rates influence the yield to a greater extent, with the cooperativity factor tipping the balance towards higher levels of folding.
5. The misfolding rate is uncorrelated with all folding efficiencies across models and cooperativity scenarios equal in folding yield. We hypothesized that these results are due to the presence of chaperones on unfolded proteins that prevent misfolding; similarly, misfolded proteins that are extracted from aggregates are stabilized by a chaperone.
6. The folding rate is correlated positively with increasing folding efficiency, although the correlation is not 1.
7. The sequestration rate is correlated negatively with folding efficiency. This result is due to two effects: (i) the loss of proteins into these insoluble structures that are not available for folding; and (ii) the entrapment of chaperones that results in lower BiP concentrations, which also has a negative effect on folding yields.

In addition to the single parameter study, we performed a variance-based global sensitivity analysis, in which we varied seven parameters (the BiP association rate, the BiP disassociation rate, the aggregation rate, the unfolding rate, the misfolding rate, the folding rate, and the sequestration rate) over two orders of magnitude simultaneously, and produced 100,000 parameter sets as input to the seven models (four non-cooperative and three cooperative models). We ran each simulation to steady state and recorded the metrics of folding efficiency and chaperone cost. From the variance-based global sensitivity analysis we learned that the sequestration rate and the aggregation rate were the dominant contributors to the variance of the output. However the variance was quite small. Our graphs then revealed for all seven parameters that the output mean across regions of parameter space was essentially constant within a model. This remarkable result indicates that the model output is rather invariant to changes in parameters. Instead our results show that model structure (the number of binding sites) and the cooperativity factor play a critical role in the behavior of the models. In addition, we also varied the concentrations of BiP and unfolded protein (U). All of these results are in Text S2, the Global Sensitivity Analysis Supplement.

### Translocation

**Figure 13:**
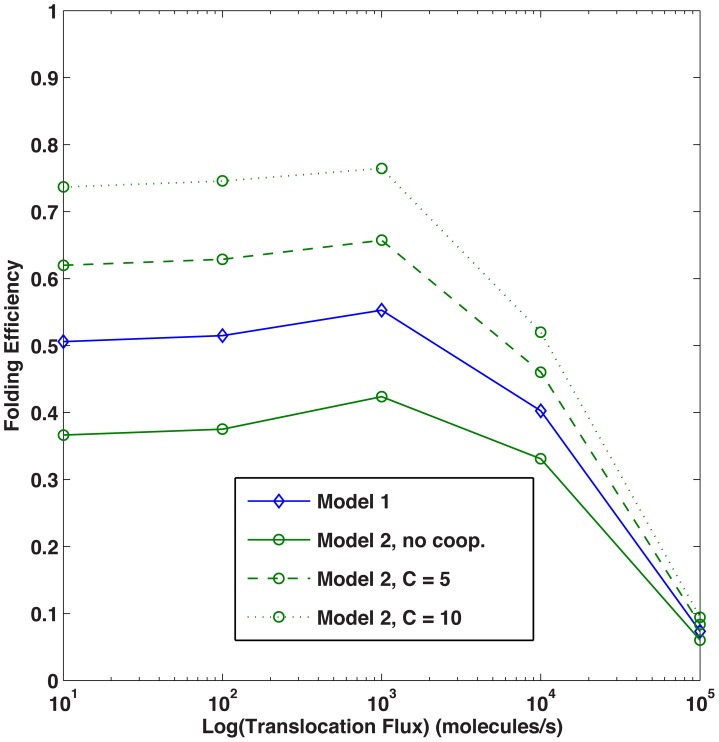
Folding efficiency vs. translocation rate. Folding efficiency as a function of translocation flux.

Finally, a translocation scenario was implemented to evaluate the impact of BiP clustering in a dynamic environment. In five different scenarios, a protein flux of 10, 100, 1000, 10000, and 100000 molecules per second was added to the ER [74] over a period of 100 seconds. Thus, many more molecules transverse the membrane to enter the ER lumen, with 10^6^ molecules initially localized in the lumen as in the steady-state case. This approach was used to mimic general ER stress in yeast. We determined that the translocation flux is highly negatively correlated (−0.96 to −0.99) with folding efficiency (Figure 13). This result was expected; as the protein flux increases, nascent proteins accumulate at the cytosol/ER membrane interface due to the limited number of pore complexes while BiP preferentially localized to ER the membrane as compared to the lumen. In the non-cooperative model, Model 1 has the highest efficiency due to the coverage effect. When cooperativity is accounted for, the multiple binding models yield a higher folding efficiency. If no unfolded proteins exist in the lumen, initially most proteins are protected and the folding efficiency is close to 1 (simulation data not shown).

## Discussion

The chaperone BiP participates in many critical ER processes, including translocation, protein folding, disaggregation, and degradation. To elucidate an improved mechanistic understanding of ER proteostasis, we constructed a computational model to evaluate ER-resident chaperone/co-chaperone interactions in which multiple BiP molecules interact with nascent and unfolded proteins to facilitate protein folding and maturation. In contrast to established models that focused only on a single site for chaperone binding events, we modeled the mechanism of entropic pulling, in which several chaperones operate in concert to unfold and disaggregate peptides by incorporating a stretching force caused by random motions of the individual chaperones. In order to investigate the acceleration of nascent proteins across an organelle membrane, entropic pulling unifies aspects of both the Brownian ratchet model [61] and power stroke model [75], [76] exceedingly well. In *S. cerevisiae*, entropic pulling was implemented successfully to track chaperone interactions during mitochondria translocation and to assess nascent proteins and aggregates [41]. Our model that incorporates this synergy represents a progress towards a mechanistic understanding of chaperone interactions.

Protein aggregation was modeled as a separate module to monitor protein fate during simulations. Results indicated that most unfolded and aggregated proteins carried out a transient interaction with chaperone molecules. Despite the stochastic binding events between BiP and unfolded proteins, the sequestration of aggregates can entrap chaperone molecules leading to decreased chaperone levels. The comparison of BiP-protein interactions, in terms of folding efficiency and levels of chaperone cost, was quantified for models containing divergent numbers of binding sites. Our results indicate that for a given concentration of BiP and proteins (*i.e.*, nascent, unfolded, or misfolded), single binding site models provided the highest degree of BiP coverage. However, experimental evidence previously showed that multiple chaperone molecules can work in concert to increase protein refolding and remove aggregates *in vivo*. Furthermore, our model revealed that the BiP-protein interaction provides additional advantages, such that multiple bound BiP molecules *prevent* misfolding of U.

Given the parameter uncertainty, we conducted a study that varied seven parameters (Figure 12) in order to examine the effects of folding efficiency in the system. Initially, each parameter was individually altered, as a global search required many sets and covered only a fraction of the parameter space. We observed that some parameters were positively correlated with folding efficiency and others were negatively correlated. The strongest effect came from the disassociation rate of BiP from unfolded proteins, because the longer the BiP could stay bound, the greater chance that folding could occur. Note: this analysis varied one parameter at a time, while keeping the others constant. Global sensitivity analysis, where all parameters were varied simultaneously, is found in the *Global Sensitivity Analysis Supplement*, Text S2.

Due to the highly conserved features between the model eukaryote, *S. cerevisiae*, and mammalian protein-folding machinery, it is extremely likely that these findings for ER translocation and protein-folding events will translate to higher eukaryotes including humans. In fact, mammalian BiP (Grp78) appears to have two functions in protein translocation: (i) it is involved in the insertion of nascent proteins into the Sec61 complex or opening of the pore itself [77], [78], and (ii) it binds to the nascent protein that laterally advances through the channel, in a manner similar to a molecular ratchet that facilitates translocation [79]–[81]. Recently, experimental studies of the mammalian homolog of the Sec complex – co-chaperone Sec63 in yeast – has been shown to recruit BiP to the translocon (*i.e.* Sec61) and activates BiP for interaction with its substrates [82], analogous to the BiP's recruitment to the translocon in yeast, as described previously. The function of many subunits of the Sec complex in mammalian cells has remained elusive due to limited experimental assessments; however, recent progress has begun to elucidate translocation efficiency, gating kinetics and functional profiling, and transport effects of subunits that comprise the mammalian Sec complex [83]–[85].

Developing spatially-relevant computational models is important as *in vivo* experiments, such as single particle tracking (SPT) and super-resolution fluorescence imaging techniques used to capture spatial effects at nanometer resolution, are relatively new technologies [86], [87]. Interestingly, under conditions of cell homeostasis BiP has been found to distribute heterogeneously throughout the yeast ER, as depicted by live cell imaging and immunofluorescence techniques [23]. In a similar manner, we conducted fluorescence spectroscopy experiments to quantify the extent that BiP gradients exist within the ER lumen (unpublished data). Under conditions of ER stress, a greater degree of BiP clustering was observed. The spatial heterogeneity of BiP is displayed via live cell imaging; in contrast, the translocation pore composed of Sec61 is distributed homogenously within the ER membrane (Figure S1, Supporting Information). Via computationally intensive efforts, and only through providing cooperative action do the advantages of clustering become evident, providing a mechanistic context for the observed differences.

In conclusion, the chaperone BiP plays several roles in the ER, namely translocation, protein folding, ER-associated degradation, and modulation of the UPR. All of these functions require that BiP perform multiple tasks to complete the process. In translocation, the accepted model is that of a Brownian ratchet, in which multiple BiP molecules bind to nascent proteins to transport them into the lumen [62], [63]. BiP's attempt to correctly fold aberrant proteins often takes multiple cycles of binding and release. We show that multiple binding facilitates aggregate dismantling through more coverage on the structures' large surfaces. In addition, our model suggests that the clustering of BiP molecules would be beneficial in terms of efficiency and chaperone cost during protein-folding processes in the ER.

## Supporting Information

Figure S1 Spatial effects of BiP and Sec61 identified by live-cell imaging. Fluorescent protein variants (*i.e.* mCherry and yEmCitrine, respectively) were fused in-frame to the C-termini of BiP and Sec61. These recombinant proteins were expressed simultaneously in haploid *S. cerevisiae* cells under the control of their endogenous promoters, as described previously [23], [88]. (A) ER-resident molecular chaperone, BiP, is localized to the nuclear and peripheral ER subcompartments. Arrows depict the heterogeneity of BiP distributed throughout the lumen, specifically within the nuclear ER. (B) In contrast, Sec61 appears to be homogeneously localized within the nuclear ER membrane, when assessed in identical cells. (C) DIC image and scale bar of 5 microns. Image was acquired by confocal microscopy (Zeiss 780 confocal microscopy, 100×/NA 1.46).
(TIF)

Text S1 Species and reactions supplement.
(PDF)

Text S2 Global sensitivity analysis supplement.
(PDF)
