# Supplementary figures and images for: BiP Clustering Facilitates Protein Folding in the Endoplasmic Reticulum
Source: PLoS Comput Biol. 2014 Jul 3;10(7):e1003675. doi: 10.1371/journal.pcbi.1003675 (PMC4081015; doi:10.1371/journal.pcbi.1003675)

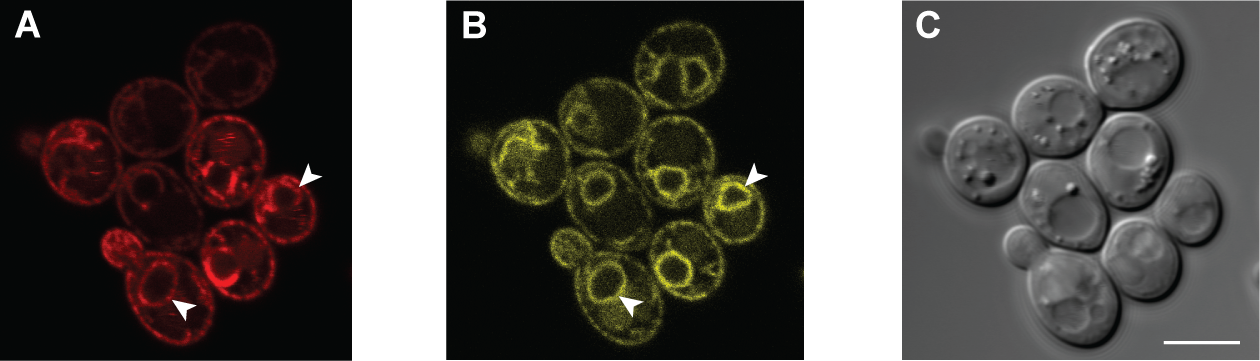

Supplement: Figure S1 — Spatial effects of BiP and Sec61 identified by live-cell imaging. Fluorescent protein variants (i.e. mCherry and yEmCitrine, respectively) were fused in-frame to the C-termini of BiP and Sec61. These recombinant proteins were expressed simultaneously in haploid S. cerevisiae cells under the control of their endogenous promoters, as described previously [23], [88]. (A) ER-resident molecular chaperone, BiP, is localized to the nuclear and peripheral ER subcompartments. Arrows depict the heterogeneity of BiP distributed throughout the lumen, specifically within the nuclear ER. (B) In contrast, Sec61 appears to be homogeneously localized within the nuclear ER membrane, when assessed in identical cells. (C) DIC image and scale bar of 5 microns. Image was acquired by confocal microscopy (Zeiss 780 confocal microscopy, 100×/NA 1.46). (TIF) [file pcbi.1003675.s001.tif]
