# Supplementary material for: BiP Clustering Facilitates Protein Folding in the Endoplasmic Reticulum
Source: PLoS Comput Biol. 2014 Jul 3;10(7):e1003675. doi: 10.1371/journal.pcbi.1003675 (PMC4081015; doi:10.1371/journal.pcbi.1003675)
Supplement: Text S1 — Species and reactions supplement. (PDF) [file pcbi.1003675.s002.pdf]

Supplement Text S1: List of Species and Reactions

Content

This file contains the species and reaction lists for the clustering model.

Species Tables

Table S1. Model Species and Initial Counts [?]

	Species	Description	Molecules
1	N	Nascent protein	0
2	Pore	Translocon	6000
3	Sec63	ER membrane co-chaperone	17700
4	Pore-Sec63	Activated pore	0
5	Pore-Sec63-N	Docked protein	0
6	BiP	Free chaperone	337000
7	Pore-Sec63-Nx	Sliding state	0
8	Pore-Sec63-N-BiP	1 bound BiP	0
9	Pore-Sec63-N-BiPx	Sliding state	0
10	Pore-Sec63-N-2BiP	2 bound BiP	0
11	Pore-Sec63-N-2BiPx	Sliding state	0
12	Pore-Sec63-N-3BiP	3 bound BiP	0
13	Pore-Sec63-N-3BiPx	Sliding state	0
14	Pore-Sec63-N-4BiP	4 bound BiP	0
15	U	Unfolded Protein	1000000
16	F	Folded Protein	0
17	M	Misfolded Protein	0
18	A2	Size 2 Aggregate	0
19	A3	Size 3 Aggregate	0
20	A4	Size 4 Aggregate	0
21	U-BiP	Chaperone-protein complex	0
22	U-2BiP	CP complex (Model 2+)	0
23	U-3BiP	CP complex (Model 3+)	0
24	U-4BiP	CP complex (Model 4)	0
25	M-BiP	Chaperone-triaged protein complex	0
26	M-2BiP	CT complex (Model 2+)	0
27	M-3BiP	CT complex (Model 3+)	0
28	M-4BiP	CT complex (Model 4)	0
29	Scj1	Lumenal co-chaperone	8260
30	U-Scj1	Co-chaperone/protein complex	0
31	U-Scj1-BiP	Chaperone-protein complex	0
32	U-Scj1-2BiP	CP complex (Model 2+)	0

continued on next page

continued from previous page

	Species	Description	Molecules
33	U-Scj1-3BiP	CP complex (Model 3+)	0
34	U-Scj1-4BiP	CP complex (Model 4)	0
35	M-Scj1	Cochaperone-triaged protein complex	0
36	M-Scj1-BiP	CM complex	0
37	M-Scj1-2BiP	CM complex (Model 2+)	0
38	M-Scj1-3BiP	CM complex (Model 3+)	0
39	M-Scj1-4BiP	CM complex (Model 4)	0
40	A2-BiP	Size 2 (Model 2+)	0
41	A2-2BiP	Size 2 (Model 2+)	0
42	A2-Scj1	Size 2 (Model 1+)	0
43	A2-Scj1-BiP	Size 2 (Model 1+)	0
44	A2-Scj1-2BiP	Size 2 (Model 2+)	0
45	A3-BiP	Size 3 (Model 1+)	0
46	A3-2BiP	Size 3 (Model 2+)	0
47	A3-3BiP	Size 3 (Model 3+)	0
48	A3-Scj1	Size 3 (Model 1+)	0
49	A3-Scj1-BiP	Size 3 (Model 1+)	0
50	A3-Scj1-2BiP	Size 3 (Model 2+)	0
51	A3-Scj1-3BiP	Size 3 (Model 3+)	0
52	A4-BiP	Size 4 (Model 1+)	0
53	A4-2BiP	Size 4 (Model 2+)	0
54	A4-3BiP	Size 4 (Model 3+)	0
55	A4-4BiP	Size 4 (Model 4+)	0
56	A4-Scj1	Size 4 (Model 1+)	0
57	A4-Scj1-BiP	Size 4 (Model 1+)	0
58	A4-Scj1-2BiP	Size 4 (Model 2+)	0
59	A4-Scj1-3BiP	Size 4 (Model 3+)	0
60	A4-Scj1-4BiP	Size 4 (Model 4+)	0

Reaction Tables

Table S2. Translocation Reactions

	Reaction	Kinetic Rate	Units	Reference
1	$\emptyset \rightarrow N$	0	Ms^{-1}	
2	$Pore + Sec63 \leftrightarrow Pore\text{-}Sec63$	10^6	$M^{-1}s^{-1}$	
3	$Pore\text{-}Sec63 + N \leftrightarrow Pore\text{-}Sec63\text{-}N$	10^6	$M^{-1}s^{-1}$	
4	$Pore\text{-}Sec63\text{-}N \leftrightarrow Pore\text{-}Sec63\text{-}Nx$	1.0	s^{-1}	
5	$Pore\text{-}Sec63\text{-}Nx + BiP \rightarrow Pore\text{-}Sec63\text{-}N\text{-}BiP$	$3.5 \cdot 10^8$	$M^{-1}s^{-1}$	[?]
6	$Pore\text{-}Sec63\text{-}N\text{-}BiP \leftrightarrow Pore\text{-}Sec63\text{-}N\text{-}BiPx$	1.0	s^{-1}	
7	$Pore\text{-}Sec63\text{-}N\text{-}BiPx + BiP \rightarrow Pore\text{-}Sec63\text{-}N\text{-}2BiP$	$3.5 \cdot 10^8$	$M^{-1}s^{-1}$	[?]
8	$Pore\text{-}Sec63\text{-}N\text{-}2BiP \leftrightarrow Pore\text{-}Sec63\text{-}N\text{-}2BiPx$	1.0	s^{-1}	
9	$Pore\text{-}Sec63\text{-}N\text{-}2BiPx + BiP \rightarrow Pore\text{-}Sec63\text{-}N\text{-}3BiP$	$3.5 \cdot 10^8$	$M^{-1}s^{-1}$	[?]
10	$Pore\text{-}Sec63\text{-}N\text{-}3BiP \leftrightarrow Pore\text{-}Sec63\text{-}N\text{-}3BiPx$	1.0	s^{-1}	
11	$Pore\text{-}Sec63\text{-}N\text{-}3BiPx + BiP \rightarrow Pore\text{-}Sec63\text{-}N\text{-}4BiP$	$3.5 \cdot 10^8$	$M^{-1}s^{-1}$	[?]
12	$Pore\text{-}Sec63\text{-}N\text{-}4BiP \rightarrow Pore\text{-}Sec63\text{-}N\text{-}3BiP + BiP$	2.0	s^{-1}	[?]
13	$Pore\text{-}Sec63\text{-}N\text{-}3BiP \rightarrow Pore\text{-}Sec63\text{-}N\text{-}2BiP + BiP$	2.0	s^{-1}	[?]
14	$Pore\text{-}Sec63\text{-}N\text{-}3BiP \rightarrow Pore\text{-}Sec63\text{-}N\text{-}2BiP + BiP$	2.0	s^{-1}	[?]
15	$Pore\text{-}Sec63\text{-}N\text{-}2BiP \rightarrow Pore\text{-}Sec63\text{-}N\text{-}BiP + BiP$	2.0	s^{-1}	[?]
16	$Pore\text{-}Sec63\text{-}N\text{-}4BiP \rightarrow Pore + Sec63 + U\text{-}4BiP$	1.0	s^{-1}	
17	$Pore\text{-}Sec63\text{-}N\text{-}3BiP \rightarrow Pore + Sec63 + U\text{-}3BiP$	1.0	s^{-1}	
18	$Pore\text{-}Sec63\text{-}N\text{-}2BiP \rightarrow Pore + Sec63 + U\text{-}2BiP$	1.0	s^{-1}	
19	$Pore\text{-}Sec63\text{-}N\text{-}BiP \rightarrow Pore + Sec63 + U\text{-}BiP$	1.0	s^{-1}	

Table S3. Aggregation Reactions

	Reaction	Kinetic Rate	Units	Reference
20	$U + U \rightarrow A2$	10^6	$M^{-1}s^{-1}$	[?, ?]
21	$M + U \rightarrow A2$	10^6	$M^{-1}s^{-1}$	[?, ?]
22	$M + M \rightarrow A2$	10^6	$M^{-1}s^{-1}$	[?, ?]
23	$A2 + U \rightarrow A3$	10^7	$M^{-1}s^{-1}$	[?]
24	$A2 + M \rightarrow A3$	10^7	$M^{-1}s^{-1}$	[?]
25	$A3 + U \rightarrow A4$	10^7	$M^{-1}s^{-1}$	[?]
26	$A3 + M \rightarrow A4$	10^7	$M^{-1}s^{-1}$	[?]
27	$A2 + A2 \rightarrow A4$	10^7	$M^{-1}s^{-1}$	[?]

Table S4. Disaggregation Reactions

	Reaction	Kinetic Rate	Units	Reference
28	$A2 + BiP \leftrightarrow A2\text{-}BiP$	$8.3 \cdot 10^5$	$M^{-1}s^{-1}$	[?]
29	$A2\text{-}BiP + BiP \leftrightarrow A2\text{-}2BiP$	$8.3 \cdot 10^5$	$M^{-1}s^{-1}$	[?]
30	$A2\text{-}BiP \rightarrow M\text{-}BiP + M$	1.0	s^{-1}	
31	$A2\text{-}2BiP \rightarrow M\text{-}BiP + M\text{-}BiP$	1.0	s^{-1}	
32	$A2 + Scj1 \leftrightarrow A2\text{-}Scj1$	$8.3 \cdot 10^5$	$M^{-1}s^{-1}$	[?]
33	$A2\text{-}Scj1 + BiP \leftrightarrow A2\text{-}Scj1\text{-}BiP$	$3.5 \cdot 10^8$	$M^{-1}s^{-1}$	[?]
34	$A2\text{-}Scj1\text{-}BiP + BiP \leftrightarrow A2\text{-}Scj1\text{-}2BiP$	$3.5 \cdot 10^8$	s^{-1}	[?]
35	$A2\text{-}Scj1\text{-}BiP \rightarrow M\text{-}Scj1 + M\text{-}BiP$	1.0	s^{-1}	
36	$A2\text{-}Scj1\text{-}2BiP \rightarrow M\text{-}Scj1\text{-}BiP + M\text{-}BiP$	1.0	s^{-1}	
37	$A3 + BiP \leftrightarrow A3\text{-}BiP$	$8.3 \cdot 10^5$	$M^{-1}s^{-1}$	[?]
38	$A3\text{-}BiP + BiP \leftrightarrow A3\text{-}2BiP$	$8.3 \cdot 10^5$	$M^{-1}s^{-1}$	[?]
39	$A3\text{-}2BiP + BiP \leftrightarrow A3\text{-}3BiP$	$8.3 \cdot 10^5$	$M^{-1}s^{-1}$	[?]
40	$A3\text{-}BiP \rightarrow A2 + M\text{-}BiP$	1.0	s^{-1}	
41	$A3\text{-}2BiP \rightarrow A2\text{-}BiP + M\text{-}BiP$	$8.3 \cdot 10^5$	$M^{-1}s^{-1}$	[?]
42	$A3\text{-}3BiP \rightarrow A2\text{-}2BiP + M\text{-}BiP$	$8.3 \cdot 10^5$	$M^{-1}s^{-1}$	[?]
43	$A3 + Scj1 \rightarrow A3\text{-}Scj1$	$8.3 \cdot 10^5$	$M^{-1}s^{-1}$	[?]
44	$A3\text{-}Scj1 + BiP \leftrightarrow A3\text{-}Scj1\text{-}BiP$	$3.5 \cdot 10^8$	$M^{-1}s^{-1}$	[?]
45	$A3\text{-}Scj1\text{-}BiP + BiP \leftrightarrow A3\text{-}Scj1\text{-}2BiP$	$3.5 \cdot 10^8$	$M^{-1}s^{-1}$	[?]
46	$A3\text{-}Scj1\text{-}2BiP + BiP \leftrightarrow A3\text{-}3BiP$	$3.5 \cdot 10^8$	$M^{-1}s^{-1}$	[?]
47	$A3\text{-}Scj1 \rightarrow A2 + M\text{-}Scj1$	1.0	s^{-1}	
48	$A3\text{-}Scj1\text{-}BiP \rightarrow A2\text{-}Scj1 + M\text{-}BiP$	1.0	s^{-1}	
49	$A3\text{-}Scj1\text{-}2BiP \rightarrow A2\text{-}Scj1\text{-}BiP + M\text{-}BiP$	1.0	s^{-1}	
50	$A3\text{-}Scj1\text{-}3BiP \rightarrow A2\text{-}Scj1\text{-}2BiP + M\text{-}2BiP$	1.0	s^{-1}	
51	$A4 + BiP \leftrightarrow A4\text{-}BiP$	$8.3 \cdot 10^5$	$M^{-1}s^{-1}$	[?]
52	$A4\text{-}BiP + BiP \leftrightarrow A4\text{-}2BiP$	$8.3 \cdot 10^5$	$M^{-1}s^{-1}$	[?]
53	$A4\text{-}2BiP + BiP \leftrightarrow A4\text{-}3BiP$	$8.3 \cdot 10^5$	$M^{-1}s^{-1}$	[?]
54	$A4\text{-}3BiP + BiP \leftrightarrow A4\text{-}4BiP$	$8.3 \cdot 10^5$	$M^{-1}s^{-1}$	[?]
55	$A4 + Scj1 \leftrightarrow A4\text{-}Scj1$	$8.3 \cdot 10^5$	$M^{-1}s^{-1}$	[?]
56	$A4\text{-}Scj1 + BiP \leftrightarrow A4\text{-}Scj1\text{-}BiP$	$3.5 \cdot 10^8$	$M^{-1}s^{-1}$	[?]
57	$A4\text{-}Scj1\text{-}BiP + BiP \leftrightarrow A4\text{-}Scj1\text{-}2BiP$	$3.5 \cdot 10^8$	$M^{-1}s^{-1}$	[?]
58	$A4\text{-}Scj1\text{-}2BiP + BiP \leftrightarrow A4\text{-}Scj1\text{-}3BiP$	$3.5 \cdot 10^8$	$M^{-1}s^{-1}$	[?]
59	$A4\text{-}Scj1\text{-}3BiP + BiP \leftrightarrow A4\text{-}Scj1\text{-}4BiP$	$3.5 \cdot 10^8$	$M^{-1}s^{-1}$	[?]
60	$A4\text{-}BiP \rightarrow A3 + M\text{-}BiP$	1.0	s^{-1}	
61	$A4\text{-}2BiP \rightarrow A3\text{-}BiP + M\text{-}BiP$	1.0	s^{-1}	
62	$A4\text{-}3BiP \rightarrow A3\text{-}2BiP + M\text{-}BiP$	1.0	s^{-1}	
63	$A4\text{-}4BiP \rightarrow A3\text{-}3BiP + M\text{-}BiP$	1.0	s^{-1}	

Table S5. Binding Reactions

	Reaction	Kinetic Rate	Units	Reference
64	$U + BiP \leftrightarrow U\text{-}BiP$	$8.3 \cdot 10^5, 2$	$M^{-1}s^{-1}, s^{-1}$	[?, ?]
65	$U\text{-}BiP + BiP \leftrightarrow U\text{-}2BiP$	$8.3 \cdot 10^5, 2$	$M^{-1}s^{-1}, s^{-1}$	[?, ?]
66	$U\text{-}2BiP + BiP \leftrightarrow U\text{-}3BiP$	$8.3 \cdot 10^5, 2$	$M^{-1}s^{-1}, s^{-1}$	[?, ?]
67	$U\text{-}3BiP + BiP \leftrightarrow U\text{-}4BiP$	$8.3 \cdot 10^5, 2$	$M^{-1}s^{-1}, s^{-1}$	[?, ?]
68	$U + Scj1 \leftrightarrow U\text{-}Scj1$	$8.3 \cdot 10^5, 2$	$M^{-1}s^{-1}, s^{-1}$	[?, ?]
69	$U\text{-}Scj1 + BiP \leftrightarrow U\text{-}Scj1\text{-}BiP$	$3.5 \cdot 10^8, 2$	$M^{-1}s^{-1}, s^{-1}$	[?, ?]
70	$U\text{-}Scj1\text{-}BiP + BiP \leftrightarrow U\text{-}2BiP$	$3.5 \cdot 10^8, 2$	$M^{-1}s^{-1}, s^{-1}$	[?, ?]
71	$U\text{-}Scj1\text{-}2BiP + BiP \leftrightarrow U\text{-}3BiP$	$3.5 \cdot 10^8, 2$	$M^{-1}s^{-1}, s^{-1}$	[?, ?]
72	$U\text{-}Scj1\text{-}3BiP + BiP \leftrightarrow U\text{-}4BiP$	$3.5 \cdot 10^8, 2$	$M^{-1}s^{-1}, s^{-1}$	[?, ?]

Table S6. Folding Reactions

	Reaction	Kinetic Rate	Units	Reference
73	$U \rightarrow F$	10^{-3}	s^{-1}	[?]
74	$U\text{-}BiP \rightarrow F + BiP$	0.1	s^{-1}	[?]
75	$U\text{-}2BiP \rightarrow F + 2BiP$	0.1	s^{-1}	[?]
76	$U\text{-}3BiP \rightarrow F + 3BiP$	0.1	s^{-1}	[?]
77	$U\text{-}4BiP \rightarrow F + 4BiP$	0.1	s^{-1}	[?]
78	$U\text{-}Scj1 \rightarrow F + Scj1$	0.1	s^{-1}	[?]
79	$U\text{-}Scj1\text{-}BiP \rightarrow F + Scj1 + BiP$	0.1	s^{-1}	[?]
80	$U\text{-}Scj1\text{-}2BiP \rightarrow F + Scj1 + 2BiP$	0.1	s^{-1}	[?]
81	$U\text{-}Scj1\text{-}3BiP \rightarrow F + Scj1 + 3BiP$	0.1	s^{-1}	[?]
82	$U\text{-}Scj1\text{-}4BiP \rightarrow F + Scj1 + 4BiP$	0.1	s^{-1}	[?]

Table S7. Misfolding/Unfolding Reactions

	Reaction	Kinetic Rate	Units	Reference
83	$U \rightarrow M$	10	s^{-1}	
84	$M + BiP \leftrightarrow M\text{-}BiP$	$8.3 \cdot 10^5$	$M^{-1}s^{-1}$	[?]
85	$M\text{-}BiP + BiP \leftrightarrow M\text{-}2BiP$	$8..3 \cdot 10^5$	$M^{-1}s^{-1}$	[?]
86	$M\text{-}2BiP + BiP \leftrightarrow M\text{-}3BiP$	$8.3 \cdot 10^5$	$M^{-1}s^{-1}$	[?]
87	$M\text{-}3BiP + BiP \leftrightarrow M\text{-}4BiP$	$8.3 \cdot 10^5$	$M^{-1}s^{-1}$	[?]
88	$M + Scj1 \leftrightarrow M\text{-}Scj1$	$8.3 \cdot 10^5$	$M^{-1}s^{-1}$	[?]
89	$M\text{-}Scj1 + BiP \leftrightarrow M\text{-}Scj1\text{-}BiP$	$3.5 \cdot 10^8$	$M^{-1}s^{-1}$	[?]
90	$M\text{-}Scj1\text{-}BiP + BiP \leftrightarrow M\text{-}Scj1\text{-}2BiP$	$3.5 \cdot 10^8$	$M^{-1}s^{-1}$	[?]
91	$M\text{-}Scj1\text{-}2BiP + BiP \leftrightarrow M\text{-}Scj1\text{-}3BiP$	$3.5 \cdot 10^8$	$M^{-1}s^{-1}$	[?]
92	$M\text{-}Scj1\text{-}3BiP + BiP \leftrightarrow M\text{-}Scj1\text{-}4BiP$	$3.5 \cdot 10^8$	$M^{-1}s^{-1}$	[?]
93	$M\text{-}BiP \rightarrow U\text{-}BiP$	1.0	s^{-1}	
94	$M\text{-}2BiP \rightarrow U\text{-}2BiP$	1.0	s^{-1}	
95	$M\text{-}3BiP \rightarrow U\text{-}3BiP$	1.0	s^{-1}	
96	$M\text{-}4BiP \rightarrow U\text{-}4BiP$	1.0	s^{-1}	
97	$M\text{-}Scj1 \rightarrow U\text{-}Scj1$	1.0	s^{-1}	
98	$M\text{-}Scj1\text{-}BiP \rightarrow U\text{-}Scj1\text{-}BiP$	1.0	s^{-1}	
99	$M\text{-}Scj1\text{-}2BiP \rightarrow U\text{-}Scj1\text{-}2BiP$	1.0	s^{-1}	
100	$M\text{-}Scj1\text{-}3BiP \rightarrow U\text{-}Scj1\text{-}3BiP$	1.0	s^{-1}	
101	$M\text{-}Scj1\text{-}4BiP \rightarrow U\text{-}Scj1\text{-}4BiP$	1.0	s^{-1}	

Table S8. Sequestering Reactions

	Reaction	Kinetic Rate	Units	Reference
102	$A2 \rightarrow I\text{-}A2$	1.0	s^{-1}	[?]
103	$A2\text{-}BiP \rightarrow I\text{-}A2\text{-}BiP$	1.0	s^{-1}	[?]
104	$A2\text{-}2BiP \rightarrow I\text{-}A2\text{-}2BiP$	1.0	s^{-1}	[?]
105	$A2\text{-}Scj1 \rightarrow I\text{-}A2\text{-}Scj1$	1.0	s^{-1}	[?]
106	$A2\text{-}Scj1\text{-}BiP \rightarrow I\text{-}A2\text{-}Scj1\text{-}BiP$	1.0	s^{-1}	[?]
107	$A2\text{-}Scj1\text{-}2BiP \rightarrow I\text{-}A2\text{-}Scj1\text{-}2BiP$	1.0	s^{-1}	[?]
108	$A3 \rightarrow I\text{-}A3$	1.0	s^{-1}	[?]
109	$A3\text{-}BiP \rightarrow I\text{-}A3\text{-}BiP$	1.0	s^{-1}	[?]
110	$A3\text{-}2BiP \rightarrow I\text{-}A3\text{-}2BiP$	1.0	s^{-1}	[?]
111	$A3\text{-}3BiP \rightarrow I\text{-}A3\text{-}3BiP$	1.0	s^{-1}	[?]
112	$A3\text{-}Scj1 \rightarrow I\text{-}Scj1$	1.0	s^{-1}	[?]
113	$A3\text{-}Scj1\text{-}BiP \rightarrow I\text{-}A3\text{-}Scj1\text{-}BiP$	1.0	s^{-1}	[?]
114	$A3\text{-}Scj1\text{-}2BiP \rightarrow I\text{-}A3\text{-}Scj1\text{-}2BiP$	1.0	s^{-1}	[?]
115	$A3\text{-}Scj1\text{-}3BiP \rightarrow I\text{-}A3\text{-}Scj1\text{-}3BiP$	1.0	s^{-1}	[?]
116	$A4 \rightarrow I\text{-}A4$	1.0	s^{-1}	[?]
117	$A4\text{-}BiP \rightarrow I\text{-}A4\text{-}BiP$	1.0	s^{-1}	[?]
118	$A4\text{-}2BiP \rightarrow I\text{-}A4\text{-}2BiP$	1.0	s^{-1}	[?]
119	$A4\text{-}3BiP \rightarrow I\text{-}A4\text{-}3BiP$	1.0	s^{-1}	[?]
120	$A4\text{-}4BiP \rightarrow I\text{-}A4\text{-}4BiP$	1.0	s^{-1}	[?]
121	$A4\text{-}Scj1 \rightarrow I\text{-}A4\text{-}Scj1$	1.0	s^{-1}	[?]
122	$A4\text{-}Scj1\text{-}BiP \rightarrow I\text{-}A4\text{-}Scj1\text{-}BiP$	1.0	s^{-1}	[?]
123	$A4\text{-}Scj1\text{-}2BiP \rightarrow I\text{-}A4\text{-}Scj1\text{-}2BiP$	1.0	s^{-1}	[?]
124	$A4\text{-}Scj1\text{-}3BiP \rightarrow I\text{-}A4\text{-}Scj1\text{-}3BiP$	1.0	s^{-1}	[?]
125	$A4\text{-}Scj1\text{-}4BiP \rightarrow I\text{-}A4\text{-}Scj1\text{-}4BiP$	1.0	s^{-1}	[?]
