# Supplementary material for: BiP Clustering Facilitates Protein Folding in the Endoplasmic Reticulum
Source: PLoS Comput Biol. 2014 Jul 3;10(7):e1003675. doi: 10.1371/journal.pcbi.1003675 (PMC4081015; doi:10.1371/journal.pcbi.1003675)
Supplement: Text S2 — Global sensitivity analysis supplement. (PDF) [file pcbi.1003675.s003.pdf]

Supplement Text S2: Global Sensitivity Analysis

For the global sensitivity analysis we ran 100,000 simulations for the 4 non-cooperative and the 3 cooperative models. We varied 7 parameters simultaneously: the BiP association rate, the BiP disassociation rate, the aggregation rate, the unfolding rate, the misfolding rate, the folding rate, and the sequestration rate. From the nominal values, each parameter was allowed to vary one order of magnitude in either direction (i.e. one-tenth of the nominal value to ten times). The folding efficiency and chaperone cost metrics were recorded at t=100 seconds, a value by which time steady-state had been established. We used a simple Monte Carlo approach to generate the random values of the input parameters independently in MATLAB. There are many more parameters in the model but we use identical values by assuming that for example the BiP association rate doesn't change whether the chaperone binds to an unfolded protein, misfolded protein, or aggregate. While future evidence may prove this not to be the case, it allowed us to test large models while keeping the model complexity down.

We used the MATLAB toolbox GUI-HDMR [1]. This toolbox uses variance-based sensitivity indices [2, 3] that quantify the relative contribution of each parameter to the uncertainties in the outputs. These methods explore the parameter space thoroughly by sampling large numbers of sets of inputs. GSA can be used to rank the importance of the various parameters as well as to discover importance interactions between parameters.

We also varied the initial conditions, though there are only two of main importance: BiP and unfolded proteins. That is because most of the large number of states in the models consist of some combination of these components and/or are derived from them. We allow those states to start at zero concentration and let the system evolve their populations during the simulation based on the reactions that occur.

Method: High Dimensional Model Representation (HDMR)

The high dimensional model representation (HDMR) is a set of tools in order to express the input-output relationship of complex models with a large number of input variables x_1,...,x_n. The output variables $f(\mathbf{x}) = f(x_1, ..., x_n)$ in the domain R^n can be written in the following form:

$$f(\mathbf{x}) = f_0 + \sum_{i=1}^{n} f_i(x_i) + \sum_{1 \geq i \geq j \geq n} f_{ij}(x_i, x_j) + ... + f_{12...n}(x_1, x_2, ..., x_n)$$

Here f_0 denotes the mean effect (zeroth order), which is a constant. The

function $f_i(x_i)$ is a first-order term giving the effect of the variable x_i acting independently (although generally non-linearly) upon the output $f(\mathbf{x})$. The function $f_{ij}(x_i, x_j)$ is a second-order term describing the interactive effects of the variables x_i and x_j upon the output $f(\mathbf{x})$. The higher-order terms reflect the effects of increasing numbers of input variables acting together to influence the output $f(\mathbf{x})$. If there is no interaction between the input variables, then only the zeroth-order term f_0 and the first-order terms $f_i(x_i)$ will appear in the HDMR expansion.

This expansion will be computationally very efficient if higher-order input variable correlations are weak and can therefore be neglected. For many systems a HDMR expression up to second-order already provides satisfactory results and a good approximation of $f(\mathbf{x})$.

In the context of global sensitivity analysis, HDMR conceptually resembles the method of Sobol [4]. In this case, the calculation is of the variance expansion of the output given N random sets of n input parameters obtained by various methods. The variance expansion function consists of partial variances of the output related to first- and second-order influences of the input parameters. That is, the total output variance of a model is the sum of contributions obtained by varying the input variables.

The total variance V can be obtained by

$$V = \int f^2(\mathbf{x})\,\mathrm{d}\mathbf{x} - f_0^2$$

and the partial variances $V_{i_1,\ldots,i_s}$ can be calculated from the HDMR expansion given above.

$$V_i = \int_0^1 f_i^2(x_i)\,\mathrm{d}x_i$$

$$V_{ij} = \int_0^1 \int_0^1 f_{ij}^2(x_i, x_j)\,\mathrm{d}x_i\,\mathrm{d}x_j$$

Once the partial variances are determined the sensitivity indices are calculated as follows:

$$S_{i_1,\ldots,i_s} = \frac{V_{i_1,\ldots,i_s}}{V}$$

where $1 \leq i_1 < \ldots < i_s \leq n$ so that all its terms add up to 1.

$$\sum_{i=1}^{n} S_i + \sum_{1 \leq i \leq j \leq n} S_{ij} + \ldots + S_{1,2,\ldots,n} = 1$$

The first-order sensitivity index S_i measures the main effect of the input variables x_i on the output, or in other words the fractional contribution of x_i to the variance of $f(\mathbf{x})$. The second-order sensitivity index S_{ij} measures the interaction effect of x_i and x_j on the output and so on.

Results

The first-order sensitivity indices are presented in Tables G1 and G2 for the output metrics folding efficiency and chaperone cost. Clearly the most important parameters of the set of seven is the sequestration parameter. This is due to the loss of chaperones due to entanglement in inclusion bodies which are inert aggregates that cannot be folded. Another parameter that is highly ranked is aggregation. The misfolding and unfolding kinetic rates have the least effect on the output for nearly all models. Surprisingly, the folding parameter has only a small effect on folding efficiency. The second-order effects were much smaller and are omitted from the tables for brevity.

Parameter	Sens	NC1	NC2	NC3	NC4	C2	C3	C4
BiPassoc	S_1	0.1229	0.0970	0.1013	0.1132	0.0430	0.0371	0.0296
BiPdisassoc	S_2	0.0199	0.0769	0.1301	0.1199	0,035	0,0245	0.0115
agg	S_3	0.1876	0.1530	0.1831	0.2086	0.042	0.0452	0.0341
unfold	S_4	0.0282	0.0134	0.023	0.0002	0.0210	0.0088	0.0032
misfold	S_5	0.0058	0.029	0.038	0.006	0.0200	0.001	0.0039
fold	S_6	0.033	0.0493	0.0610	0.0702	0.0812	0.1083	0.0993
seq	S_7	0.5607	0.5034	0.4561	0.3736	0.6871	0.7622	0.8111
sum	$\sum S_i$	0.9581	0.9220	0.9926	0.8917	0.9293	0.9871	0.9927

Table G1. First-order sensitivity indices for all models (folding efficiency).

Parameter	Sens	NC1	NC2	NC3	NC4	C2	C3	C4
BiPassoc	S_1	0.0465	0.2070	0.0760	0.1141	0.0612	0.0569	0.0249
BiPdisassoc	S_2	0.0222	0.1248	0.2299	0.1166	0.058	0.1010	0.0130
agg	S_3	0.2692	0.0837	0.0942	0.2080	0.1581	0.0648	0.0349
unfold	S_4	0.0013	0.0001	0.0110	0.008	0.033	0.0002	0.0125
misfold	S_5	0.0009	0.0002	0.009	0.012	0.012	0.001	0.001
fold	S_6	0.012	0.0003	0.0356	0.0632	0.021	0.0054	0.1013
seq	S_7	0.5849	0.4799	0.4737	0.4214	0.6417	0.7655	0.809
sum	$\sum S_i$	0.937	0.896	0.9294	0.9433	0.9825	0.9948	0.9966

Table G2. First-order sensitivity indices for all models (chaperone cost).

Global sensitivity analysis does not measure the absolute size of the output variance, just the relative contributions to it. Thus, we examined the means and variances of all models for the metrics of chaperone cost and folding efficiency.

Means and variances of the output due to varying input parameters

The means of the folding efficiency metric for the non-cooperative scenarios confirm that the single binding site model produces the most folding (Table G3). This is due to the coverage effect as there are fewer binding sites

to saturate on unfolded proteins and prevent them from misfolding. However, the cooperativity of multiple BiPs acting in concert through entropic pulling increases the amount of folding over the single binding site model. Thus, our large parameter study confirms our basic findings. In terms of chaperone cost, the non-cooperative models take a larger fraction of total chaperones per unit time to do their folding work. However, when cooperativity is introduced into the model, the chaperone cost of the higher binding site models drops below the single binding site model (Table G4). More chaperones are still bound to unfolded, misfolded, and aggregated proteins, but with the higher kinetic rate introduced by cooperativity, the speed of their action lowers the effective chaperone cost. Thus, our findings with the nominal parameters were confirmed by the large set of parameter values.

model	NC1	NC2	NC3	NC4	C2	C3	C4
mean	0.1904	0.1031	0.0766	0.0795	0.3188	0.2831	0.2649
variance	0.0159	0.0056	0.0042	0.0037	0.0291	0.0253	0.0265

Table G3. Mean and variance for all models for the folding efficiency metric.

model	NC1	NC2	NC3	NC4	C2	C3	C4
mean	1.211	1.675	1.746	1.842	0.203	.245	0.3021
variance	0.0042	0.0162	0.0172	0.0218	0.0208	0.0322	0.0158

Table G4. Mean and variance for all models for the chaperone cost metric.

In fact, the variance tells a very important story. Over 700 thousand parameter sets, the output is quite invariant to changes in these inputs. Instead the results are a consequence of model structure. For each parameter, we rescaled the values from 0 to 1 and divided the input space into tenths (We call this the input percentile). From that restructuring, we calculated the mean of the outputs for the simulations for each of the 10 bins (folding efficiency and chaperone cost). We found that regardless of the region of the single parameter input space, the mean output metric was nearly constant. This was remarkable because one would think that the gulf from the 10th to the 90th percentile with a parameter like sequestration would produce different outputs. But the output was quite invariant. This was true for all seven parameters tested; the small variance in the output metrics for all models validates this.

Folding Efficiency Metric for All Models

Figures G1-G7 show the folding efficiency for the cooperative and non-cooperative models.We defined the input percentile as the folding efficiency mean of each of ten bins. The basic results for folding efficiency were not different from the nominal parameters, but it did show that these results did not depend on parameters at all. We suspect that the number of binding sites (model structure), the coverage effect, and the cooperativity were the main causes of the different folding efficiencies in line with the main results.

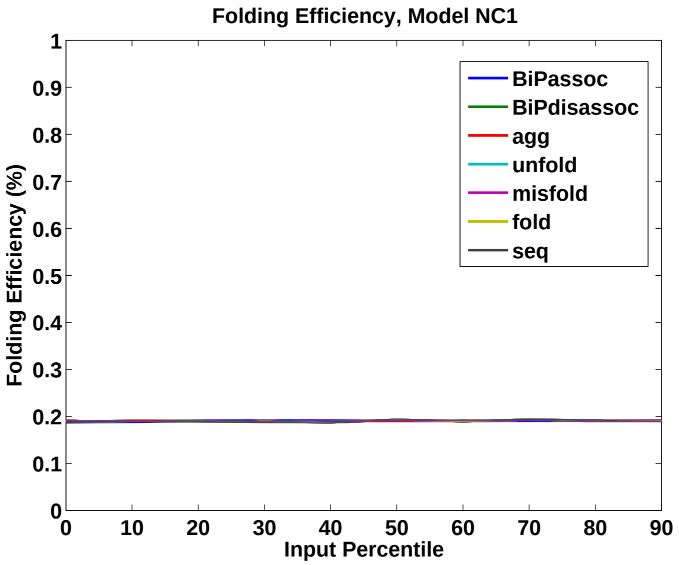

Figure G1. Folding efficiency of model NC1 as a function of input parameters.

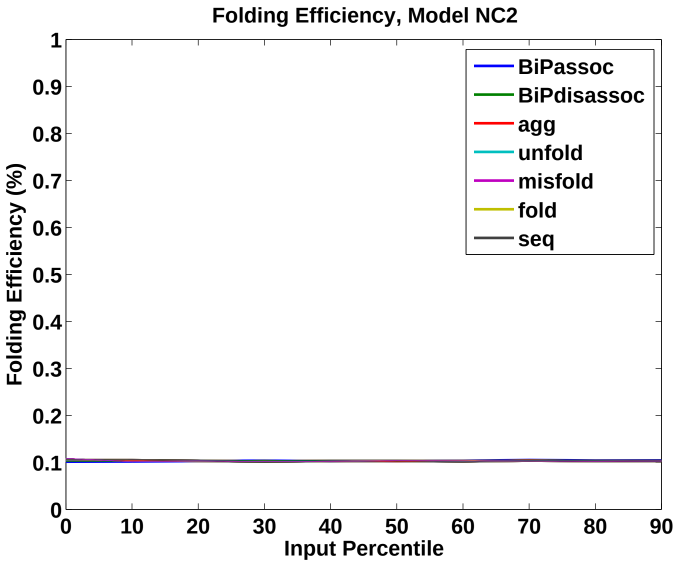

Figure G2. Folding efficiency of model NC2 as a function of input parameters.

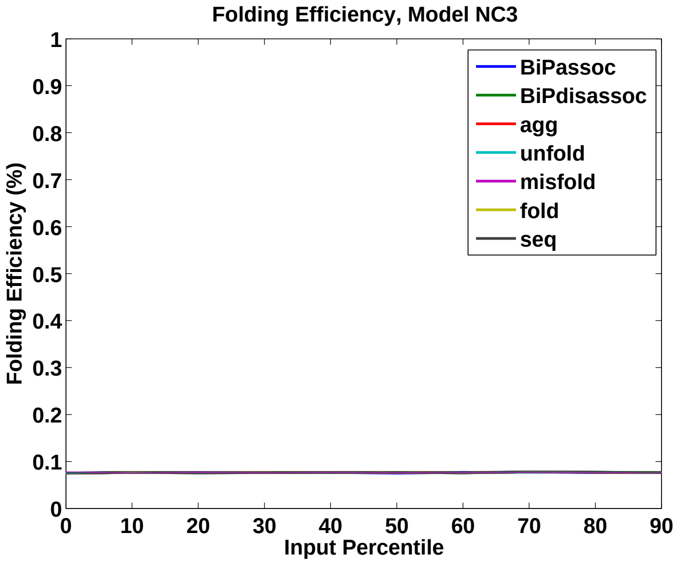

Figure G3. Folding efficiency of model NC3 as a function of input parameters.

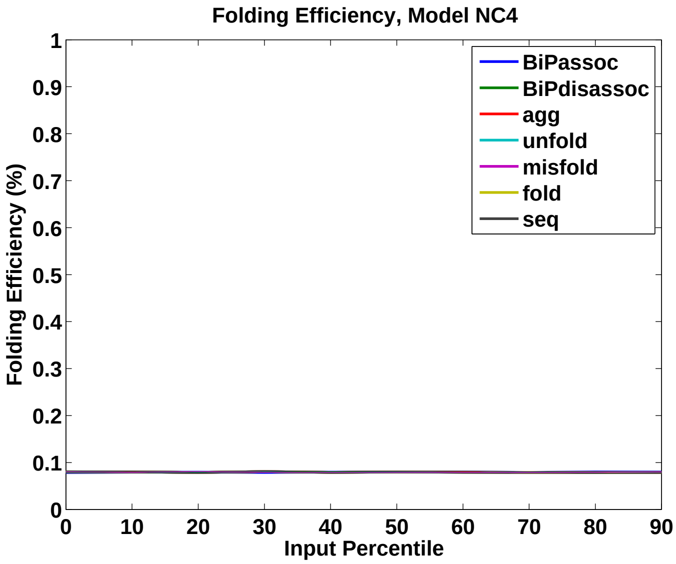

Figure G4. Folding efficiency of model NC4 as a function of input parameters.

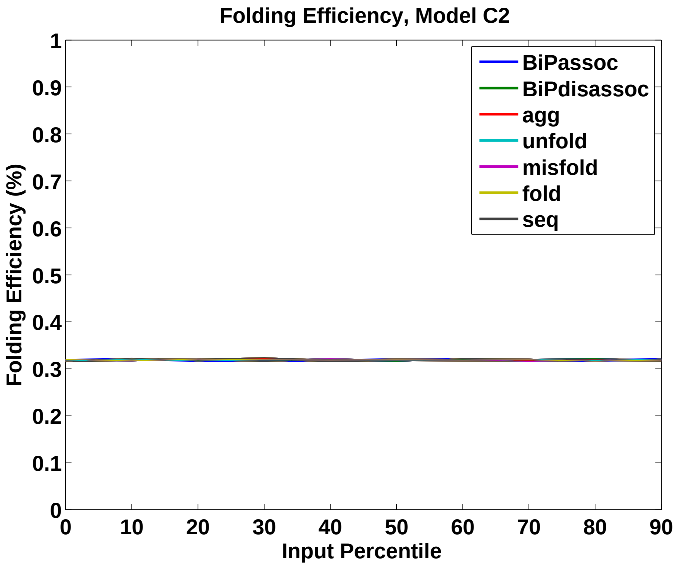

Figure G5. Folding efficiency of model C2 as a function of input parameters.

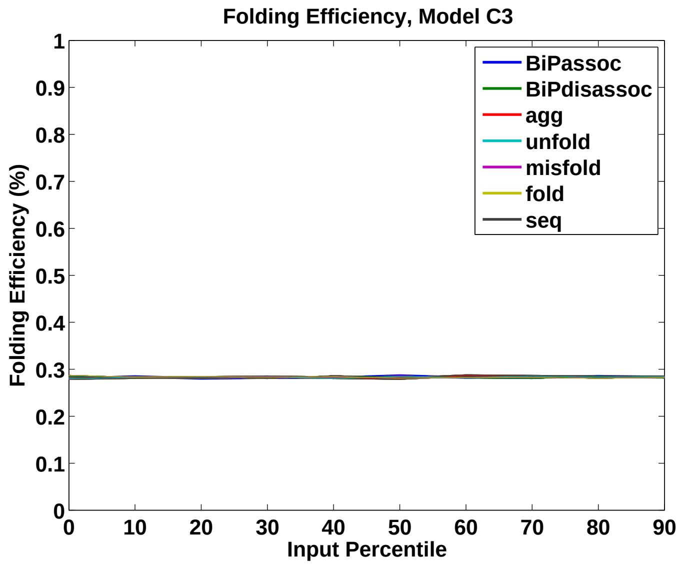

Figure G6. Folding efficiency of model C3 as a function of input parameters.

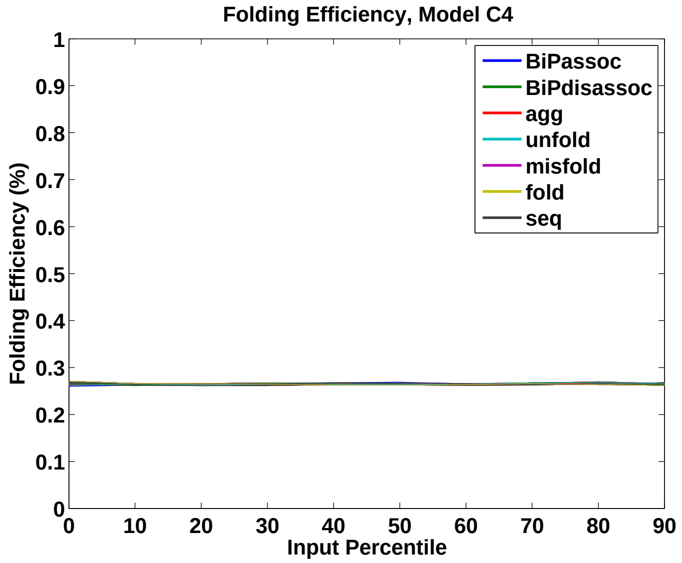

Figure G7. Folding efficiency of model C4 as a function of input parameters.

Cooperative Effects on Folding Efficiencies

Figures G8-G10 show the ratio of folding efficiencies for a cooperative model compared to its non-cooperative counterpart for cooperativity parameter $C = 10$. As the figures demonstrate, there is a several fold increase in folding efficiency, with very little variance in the findings across input space. The effect is even greater for models 3 and 4 compared 2, because their non-cooperative versions have a lower folding efficiency due to the coverage effect. This confirms our original findings that cooperativity through the concerted action of multiple BiPs is a major effect of our simulations.

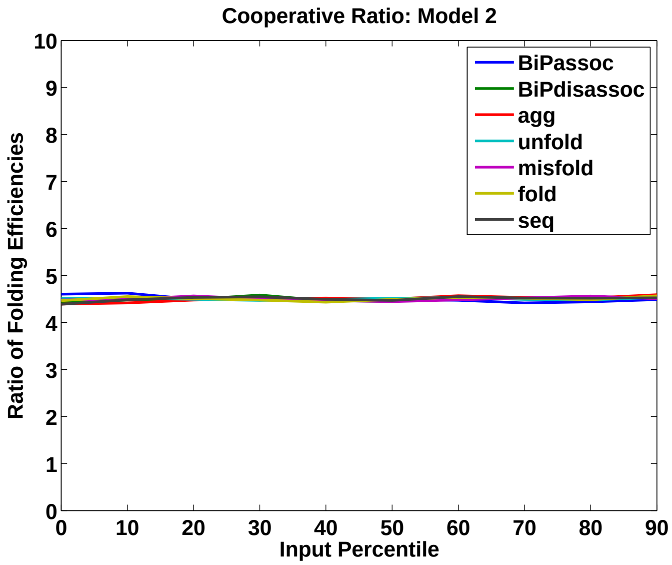

Figure G8. Effect of cooperativity on model 2 folding efficiencies as a function of input parameters.

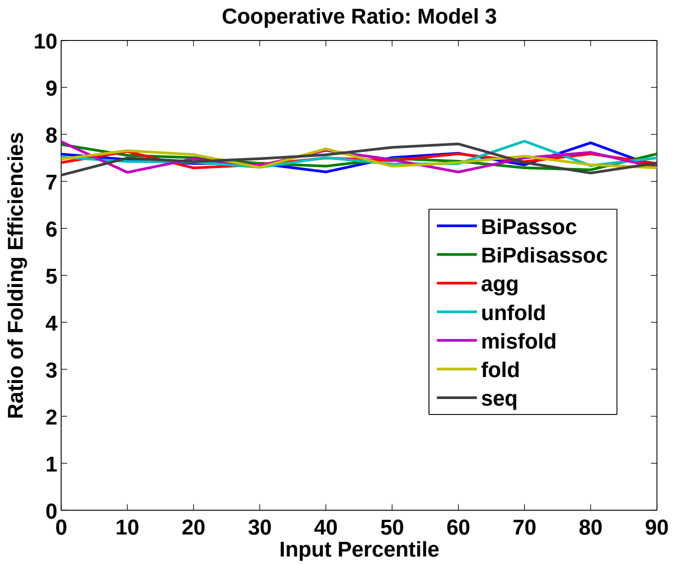

Figure G9. Effect of cooperativity on model 3 folding efficiencies as a function of input parameters.

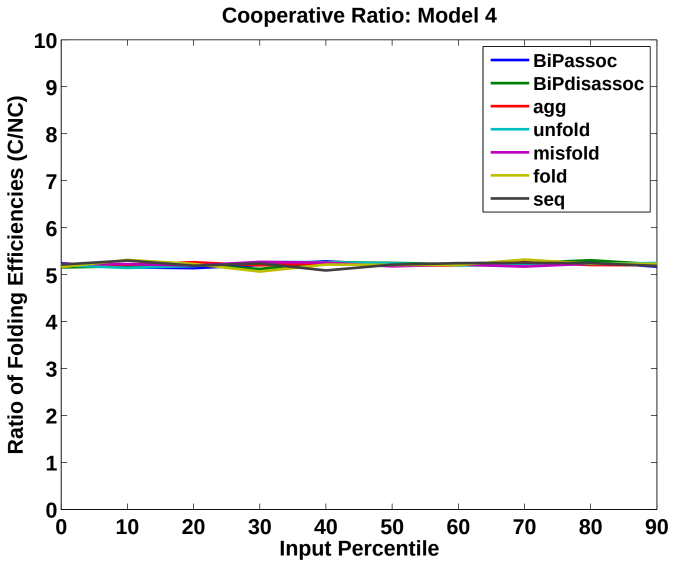

Figure G10. Effect of cooperativity on model 4 folding efficiencies as a function of input parameters.

Effect on Chaperone Cost Ratio of Varying Input Parameters

Figures G11-G13 (referring to models 2, 3, and 4, respectively) confirm our findings that chaperone cost drastically decreases when cooperativity is introduced into the model. In fact, the metric is less than a tenth of the cost for the comparative non-cooperative model. This ratio is quite invariant across the space of the 7 parameter values tested.

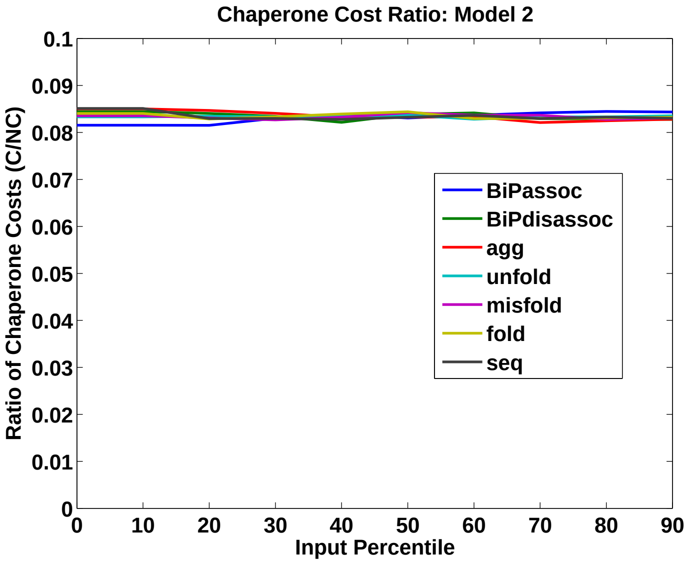

Figure G11. Effect of cooperativity on model 2 chaperone costs as a function of input parameters with a cooperativitiy parameter of 10.

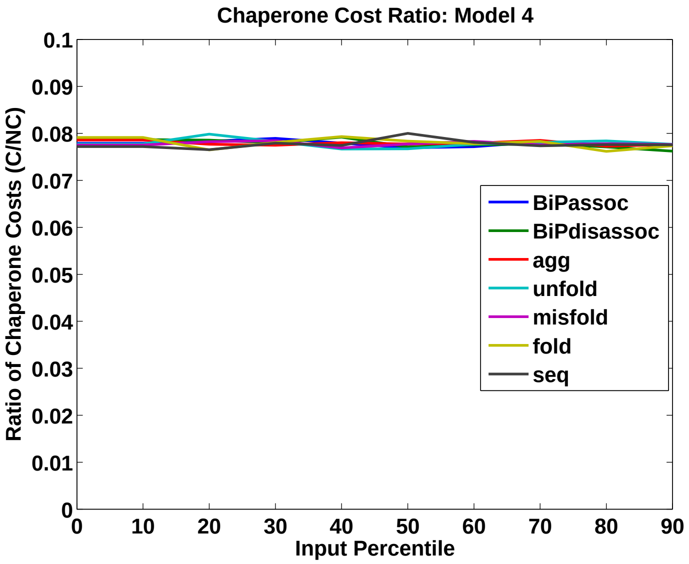

Figure G12. Effect of cooperativity on model 3 chaperone costs as a function of input parameters with a cooperativitiy parameter of 10.

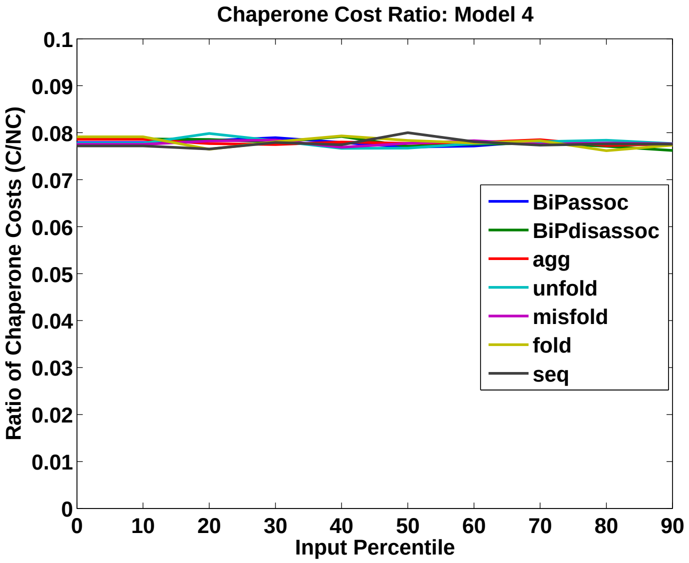

Figure G13. Effect of cooperativity on model 4 chaperone costs as a function of input parameters with a cooperativitiy parameter of 10.

Variation of the Amount of BiP and U Initial Conditions and Its Effect on Folding Efficiency

We also varied the amount of BiP and U molecules in the simulations' initial conditions simultaneously. Each species had a range of $100,000 - 10^6$ (equivalent concentrations), incremented by 100,000 molecules for a total number of 100 simulations for each of the seven models. We measured the folding efficiency metric as a function of the two initial conditions. Figure G14 shows heatmap plots for each of the 7 models. For the non-cooperative models, the single binding site case produced the most folding over the range compared to models 2,3, and 4. This confirms our previous finding that the coverage effect caused the binding sites to be more saturated for model 1, leading to more folding and prevention of misfolding and aggregation.

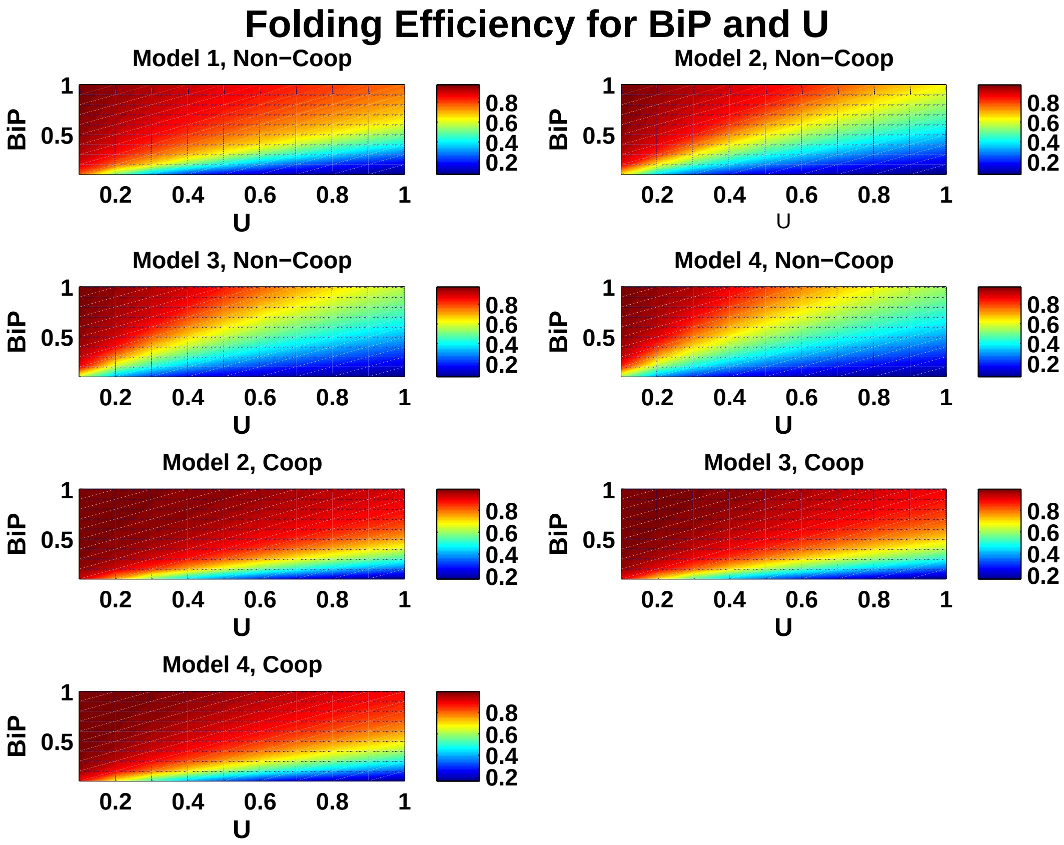

Figure G14. Effect of varying BiP and U simultaneously on folding efficiency for all models.

References

1. Ziehn T, Tomlin A (2009) GUI-HDMR- a software tool for global sensitivity analysis of complex models. Environmental Modeling and Software 24: 775-785.

2. Zhang X, Pandey M (2014) An effective approximation for variance-based global sensitivity analysis. Reliability Engineering and System Safety 121: 164-174.

3. Wainwright H, Finsterle S, Jung Y, Zhou Q, Birkholzer J (2014) Making sense of global sensitivity analyses. Computers and Geosciences 65: 84-94.

4. Sobol I (2001) Global sensitivity indices for nonlinear mathematical models and their Monte Carlo estimates. Mathematics and Computers in Simulation 55: 271-280.
